# Association between SNPs within candidate genes and compounds related to boar taint and reproduction

**DOI:** 10.1186/1471-2156-10-32

**Published:** 2009-07-05

**Authors:** Maren Moe, Sigbjørn Lien, Torunn Aasmundstad, Theo HE Meuwissen, Marianne HS Hansen, Christian Bendixen, Eli Grindflek

**Affiliations:** 1 The Norwegian Pig Breeders Association (NORSVIN), Hamar, Norway; 2 Department of Animal and Aquacultural Sciences, Norwegian University of Life Sciences, Ås, Norway; 3 Centre for Integrative Genetics (CIGENE), Norwegian University of Life Sciences, Ås, Norway; 4 Faculty of Agricultural Sciences, University of Aarhus, Tjele, Denmark

## Abstract

**Background:**

Boar taint is an unpleasant odour and flavour of the meat from some uncastrated male pigs primarily caused by elevated levels of androstenone and skatole in adipose tissue. Androstenone is produced in the same biochemical pathway as testosterone and estrogens, which represents a particular challenge when selecting against high levels of androstenone in the breeding programme, without simultaneously decreasing levels of other steroids. Detection of single nucleotide polymorphisms (SNPs) associated with compounds affecting boar taint is important both for gaining a better understanding of the complex regulation of the trait and for the purpose of identifying markers that can be used to improve the gain of breeding. The beneficial SNPs to be used in breeding would have the combinational effects of reducing levels of boar taint without affecting fertility of the animals. The aim of this study was to detect SNPs in boar taint candidate genes and to perform association studies for both single SNPs and haplotypes with levels of boar taint compounds and phenotypes related to reproduction.

**Results:**

An association study involving 275 SNPs in 121 genes and compounds related to boar taint and reproduction were carried out in Duroc and Norwegian Landrace boars. Phenotypes investigated were levels of androstenone, skatole and indole in adipose tissue, levels of androstenone, testosterone, estrone sulphate and 17β-estradiol in plasma, and length of bulbo urethralis gland. The SNPs were genotyped in more than 2800 individuals and several SNPs were found to be significantly (LRT > 5.4) associated with the different phenotypes. Genes with significant SNPs in either of the traits investigated include cytochrome P450 members *CYP2E1*, *CYP21*, *CYP2D6 *and *CYP2C49*, steroid 5α-reductase *SRD5A2*, nuclear receptor *NGFIB*, catenin *CTNND1*, BRCA1 associated protein *BAP1 *and hyaluronoglucosaminidase *HYAL2*. Haplotype analysis provided additional evidence for an effect of *CYP2E1 *on levels of skatole and indole, and for *BAP1*, *HYAL2 *and *SRD5A2 *on levels of androstenone.

**Conclusion:**

The findings in this study indicate that polymorphisms in *CYP2E1, CYP21, CYP2D6, CYP2C49, NGFIB *and *CTNND1 *might be used to reduce levels of boar taint without affecting levels of testosterone, estrone sulphate, 17β-estradiol or length of bulbo urethralis gland.

## Background

Male pigs used for meat production are castrated at an early age to avoid boar taint, which is an unpleasant odour and flavour of the meat from some boars. Due to animal welfare concerns, castration will be prohibited in Norway, and possibly EU countries and others. Alternative methods are therefore needed to prevent tainted meat. Because of available testicular steroids, entire male pigs also have better feed conversion and carcass traits compared to barrows and this makes them more advantageous for the pig industry [1]. Reduction of boar taint levels without castration is therefore of interest for pig breeders worldwide. Identification of genetic factors controlling boar taint may be implemented in breeding programmes to select animals that produce low levels of taint. However, selection for low boar taint generally coincides with selection for low androgen production [2]. Before starting selection it is therefore important to understand the complex genetic system controlling boar taint and to take into account possible correlated effects on other traits in the breeding goal.

Boar taint is mainly caused by elevated levels of the compounds androstenone [3] and/or skatole [4] in adipose tissue. Androstenone (5α-androst-16-en-3-one) is a 16-androstene steroid metabolised from cholesterol through the C_21 _steroids pregnenolone and progesterone in boar testis [5]. It is further reduced to its alcohols α-androstenol and β-androstenol [6], which also, in a lesser degree, contribute to tainted meat [7]. Skatole (3-methylindole) is a metabolite of the amino acid tryptophan and is produced by intestinal bacteria in the gut [8,9]. Skatole is only a problem in intact male pigs and not in gilts or barrows, and its levels increase at sexual maturity [10,11]. Indole is another metabolite from tryptophan. It also contributes to boar taint levels, although to less extent than androstenone and skatole [12]. Both androstenone and skatole are degraded in the liver and a relationship between their metabolism has been found [13].

Pregnenolone and progesterone are not only precursors of androstenone, but also of testosterone and estrogens [14]. The influence of other sex steroids on levels of androstenone and skatole has been studied with ambiguous results. Most studies have found levels of testosterone in plasma not to be correlated to levels of androstenone in adipose tissue [13,15,16] or levels of skatole in adipose tissue [13,15,17,18]. In some studies, however, levels of androstenone in adipose tissue [18,19] have been found to be correlated (0.26 – 0.64) to levels of testosterone. Results for estrogens are more consistent, showing positive correlations to levels of both androstenone (0.42 – 0.93) [13,15,16,18-20] and skatole (0.29 – 0.53) [13,15,17,20] in adipose tissue. No correlation between levels of skatole and estrone sulphate was, however, found in one study [18]. The correlations between levels of androstenone in plasma and adipose tissue diverge from high (0.46–0.94) [18,19,21-23] to not significant [24,25]. Levels of skatole and indole in adipose tissue are shown to be highly correlated (0.46–0.75) [26,10]. Studies on correlations between levels of androstenone and skatole in adipose tissue show inconsistent results, from medium correlations around 0.3 [18,27,28] to higher correlations between 0.45 and 0.68 [15,23]. Levels of androstenone in plasma has been found correlated (0.44) [13] and not correlated [18,23] to levels of skatole, while levels of skatole in plasma has been shown correlated (0.76) [23] and not correlated [18] to levels of androstenone in adipose tissue. Diverging results might be explained by breed effects, which significantly affect levels of boar taint, e.g. [29]. Moreover, levels of androstenone, testosterone and estrogens in plasma can also be affected by diurnal variations during the day, e.g. [30,25,31].

Detection of single nucleotide polymorphisms (SNPs) associated with boar taint compounds may be applied in practical breeding to reduce boar taint in intact boars. However, before implementation, it is of importance to examine associations of these SNPs with phenotypes related to reproduction because of possible unfavourable correlations. Breed specific variations has been observed for both androstenone [29] and skatole [10] and it is therefore of interest to include different breeds in such an examination. The objective of this study was to genotype candidate gene SNPs in Duroc and Norwegian Landrace boars and to study associations with traits related to boar taint and reproduction. The phenotypic traits included were levels of androstenone, skatole and indole in adipose tissue, levels androstenone, testosterone, 17β-estradiol and estrone sulphate in plasma, and the length of bulbo urethralis gland. Estrogens and the length of bulbo urethralis gland are also indicators of sexual maturity, e.g. [32,17,33]. Moreover, haplotype analyses were carried out in genes with several SNPs.

## Methods

### Animals

A total of 1102 Duroc and 1726 Norwegian Landrace boars were included in this study. The boars were the sons of 81 Duroc and 90 Norwegian Landrace sires. The boars were raised at NORSVIN's boar test stations until 100 kg live weight, on average 156 and 143 days for Duroc and Norwegian Landrace, respectively, and slaughtered on average 15 days later. The days between 100 kg live weight and slaughter are due to the boar selection process, where some animals wait for their destiny as elite boars. Blood samples were taken three days before slaughter for plasma suspension and DNA extraction. Samples from subcutaneous fat were taken at the slaughter line. All the samples were stored at -20°C until chemical analyses or DNA extraction was performed. The length of bulbo urethralis gland was measured at the slaughter line.

### Chemical analyses

Levels of androstenone, skatole and indole in adipose tissue and plasma were analysed at the hormone laboratory at the Norwegian School of Veterinary Sciences (NVH). Levels of androstenone were analysed by a modified time-resolved fluoroimmunoassay [34], using an antibody produced at NVH [35], whereas levels of skatole and indole in adipose tissue were analysed using high performance liquid chromatography [26]. Levels of testosterone, 17β-estradiol and estrone sulphate in plasma were analysed at the hormone laboratory at Aker University Hospital. Plasma levels of testosterone were measured by a radioimmunoassay (Orion Diagnostica, Espoo, Finland). The intra- and total assay coefficients of variation (CVs) were 7% and 9%, respectively. Plasma levels of estradiol were measured by a fluoroimmunoassay (Perkin Elmer, Turku, Finland). The intra- and total assay coefficients of variation (CVs) were 3% and 7%, respectively. Plasma levels of estrone sulphate were measured by a radioimmunoassay (Diagnostic System Laboratories, Inc., Webster, TX, USA). The intra- and total assay coefficients of variation (CVs) were 5% and 7%, respectively.

### DNA extraction

DNA was isolated from leukocytes using the automated DNA extractor Bio Robot M48 from Qiagen (CA, USA) and the supplementary MagAttract DNA Blood Midi M48 protocol. Concentrations were measured on a 1420 Victor plate reader (Turku, Finland) using PicoGreen fluorescence (Molecular Probes, OR, USA) or on a NanoDrop ND-1000 Spectrophotometer (NanoDrop Technologies, DE, USA). Normalisation of DNA samples was done using the Biomek FX robot from Beckman (Beckman Coulter, CA, USA).

### SNP discovery and validation

On the basis of their known or putative role in boar taint, candidate genes were chosen based on literature studies and on results from our previously published microarray results [36,37]. SNP discovery was performed by PCR resequencing of genomic DNA and cDNA from Duroc and Norwegian Landrace boars. Primers were designed using Primer3 [38] or Oligo primer analysis software v.6 (Molecular Biology Insights, Inc., CO, USA). The programmes Phred, Phrap and PolyPhred (v.4.06) were used to identify putative SNPs from the PCR resequencing chromatograms [39,40] and the Consed programme was used to visually confirm the putative SNPs [41]. Additionally, SNPs were provided from alignment of EST sequences produced in the Sino-Danish sequencing project [42]. The genotyping was done in a two-step approach. Before high throughput genotyping, all the SNPs (a total of 275 SNPs in 121 genes) were validated on 380 animals from each breed using the genotyping procedure described below (see additional file 1: Genotyped SNPs). The SNPs that were significant in at least one of the phenotypes using a non-stringent significance level (p < 0.1) were used for genotyping of all the boars. Of the selected SNPs, 135 were in successful assays (see additional file 1: Genotyped SNPs).

### Genotyping

SNPs were genotyped using matrix-assisted laser desorption/ionisation time-of-flight mass spectroscopy (MALDI-TOF MS) assays. Multiplex assays for use in the Sequenom MassARRAY system were designed using MassARRAY Assay Design software (Sequenom, San Diego, USA) at multiplexing levels between 7 and 35. Primers for the genotyping can be found in additional file 2: Primer sequences for genotyping. Genotyping was done by the IPLEX protocol using manufacturer's instructions (Sequenom, San Diego, USA) [43]. The MassARRAY Typer software was used for automated genotype calling.

### Haplotype construction

Genes with more than one SNP were used for haplotype analyses if one of the SNPs was significant for one of the phenotypes. Haplotype construction and frequency estimation were done using two programmes in combination. CRIMAP v2.4 [44] used pedigree information while PHASE v2.1.1 [45,46] used linkage disequilibrium information to determine haplotypes.

### Statistical analyses

Statistical analyses were performed for the two breeds Duroc and Norwegian Landrace separately. Association studies were done using the likelihood ratio test [47] by average information restricted maximum likelihood (AI-REML) [48] combined with expectation maximisation (EM-REML) if an update goes outside parameter space [49]. The procedure is part of the package DMU, v.6 release 4.7 [47]. The fixed effects fitted were sire, herd-year-season, waiting in boar test station before slaughter or not, and pen. By having sire as a fixed effect we make sure that the sire family effect does not affect the SNP estimate. Covariates used were age at 25 kg (start of boar test), days from 25 kg to 100 kg (days in boar test), days from 100 kg to slaughter (days in waiting station) and number of live born in same litter. Number of live born was also included as a squared term as this was shown to have a significant effect in the model. Animal ID, sample date for adipose tissue or plasma, and SNP-genotype or haplotype-genotype were fitted as random effects. The model used was as follows



where trait is bulbo urethralis gland length (cm), ln(ppm levels of androstenone in adipose tissue), ln(ppm levels of skatole in adipose tissue), ln(ppm levels of indole in adipose tissue), ln(ppm levels of androstenone in plasma), ln(ppm levels of testosterone in plasma), ln(ppm levels of estrone sulphate in plasma) or ln(ppm levels of 17β-estradiol in plasma). The linear model used assumes normality. The distributions were, however, skewed for all the chemical compounds in the study and the data was log-transformed to reduce this problem.

In the haplotype analyses, SNP was replaced by haplotype. SNPs with a genotyping success rate of less than 90% were excluded from further analyses and missing data was considered as a separate class in the random effect of SNP or haplotype. For each trait the model was run without SNPs or haplotypes for log likelihood comparison. A log likelihood ratio (LRT) exceeding 5.4 units, corresponding p < 0.001, was considered significant (assuming that 2*LRT is approximately chi-squared distributed with one degree of freedom).

## Results

A total of 135 SNPs from 57 candidate genes for boar taint were included in this study. The SNPs were distributed in exons, introns and untranslated regions of the genes and were genotyped in the two breeds Duroc (D) and Norwegian Landrace (NL). Out of these, 9 and 4 SNPs were monomorphic in D and NL, respectively (Table 1). SNPs with an estimated minor allele frequency (MAF) of less than 1% were excluded from further analyses. For the D breed, 19 SNPs were excluded due to low MAF while for NL this number was 14 (Table 1). The resulting SNPs with a genotyping success rate of more than 90% were used for association studies. A summary of these SNPs and their genes, alleles and frequencies are presented for D in Table 2 and for NL in Table 3. Descriptive statistics for the different phenotypes used in association analyses are presented for D in Table 4 and for NL in Table 5. Significant effects of SNPs on these phenotypes are shown in Table 6.

**Table 1:** SNPs excluded from further analysis due to low variation.

Breed	Fixed SNPs
D	ALB_1103(ex9), AKR1C3_in4d, CRSP9_504(ex1), FTH1_3'UTR, MMP1_in3a, RALBP1_3'UTR, HSP70_1748(ex1), HSP70_1258(ex1)

NL	CYP21_in8c, HSD17B4_in18d, HSP70_939(ex1), HSP70_1476(ex1)

	Estimated MAF < 0.01

D	AKR1C3_in2a, AKR1C3_in4a, BAP1_3'UTRb, CYP21_in6a, CYP21_in8a, CYP2E1_1422(ex9), EGFR_3'UTRa, EGFR_in12, HBLD2_3'UTRa, HBLD2_3'UTRb, HSPCA_3'UTR, HSPCA_2175(ex9), HYAL1_75(ex1), HYAL2_in1b, MMP13_in2, MMP13_in3a, PAPSS2_3'UTRa, PIAS1_1863(ex14), SRD5A2_3'UTRc
NL	ATP5F1_183(ex3), AKR1C3_in2a, FTH1_5'UTR, HBLD2_3'UTRb, HBS1L_1994(ex17), HYAL1_83(ex1), MMP1_279(ex2), NGFIB_1195(ex4), NGFIB_in4, NGFIB_1374(ex5), PAPS2_3'UTRb, UGT1A1_in3a, UGT1A1_in3b, UGT2B17_197(ex1)

Fixed SNPs and SNPs with an estimated minor allele frequency (MAF) of less than 1% were not included in the association study.

**Table 2:** SNPs used for the final association analyses in Duroc.

**SNP ID**	**Alleles**	**MAF**	**Homozygote 1**	**Heterorozygote**	**Homozygote 2**
AK1_483(ex5)	(C) T	0.176	C/C (n = 41)	C/T (n = 290)	T/T (n = 725)
AKR1C3_in4b	C (T)	0.436	C/C (n = 331)	C/T (n = 518)	T/T (n = 198)
AKR1C3_in4c	C (T)	0.449	C/C (n = 302)	C/T (n = 501)	T/T (n = 199)
ATP5F1_183(ex3)	(A) G	0.104	A/A (n = 9)	A/G (n = 204)	G/G (n = 855)
Bap1_3'UTRa	(A) T	0.055	A/A (n = 2)	A/T (n = 112)	T/T (n = 947)
CTNND1_3'UTRa	(G) T	0.11	G/G (n = 13)	G/T (n = 199)	T/T (n = 811)
CTNND1_3'UTRb	(A) G	0.104	A/A (n = 14)	A/G (n = 186)	G/G (n = 825)
CYB5_-8(prom)	G (T)	0.028	G/G (n = 995)	G/T (n = 60)	-
CYP11B1_in1a	(C) G	0.015	C/C (n = 2)	C/G (n = 29)	G/G (n = 1036)
CYP21_in9	(A) G	0.075	A/A (n = 11)	A/G (n = 135)	G/G (n = 905)
CYP21_in6b	(C) T	0.075	C/C (n = 11)	C/T (n = 137)	T/T (n = 919)
CYP21_in8b	A (G)	0.075	A/A (n = 893)	A/G (n = 134)	G/G (n = 11)
CYP21_in8c	C (T)	0.276	C/C (n = 554)	C/T (n = 438)	T/T (n = 75)
CYP21_in8d	(A) C	0.322	A/A (n = 122)	A/C (n = 415)	C/C (n = 485)
CYP2C49_1083(ex7)	C (G)	0.499	C/C (n = 2)	C/G (n = 1041)	G/G (n = 1)
CYP2D6_1276(ex7)	C (T)	0.228	C/C (n = 642)	C/T (n = 382)	T/T (n = 55)
CYP2D6_1287(ex7)	(A) G	0.148	A/A (n = 21)	A/G (n = 260)	G/G (n = 739)
CYP2E1_1423(ex9)	(A) G	0.48	A/A (n = 244)	A/G (n = 489)	G/G (n = 285)
CYP2E1_in1a	(C) G	0.296	C/C (n = 98)	C/G (n = 1420)	G/G (n = 521)
CYP2E1_in1b	C (DEL)	0.482	C/C (n = 287)	C/D (n = 545)	D/D (n = 248)
CYP2E1_in6	C (T)	0.482	C/C (n = 284)	C/T (n = 536)	T/T (n = 246)
CYP3A4_1498(ex13)	(A) G	0.497	A/A (n = 232)	A/G (n = 566)	G/G (n = 238)
EGFR_3'UTRb	(C) T	0.164	C/C (n = 28)	C/T (n = 304)	T/T (n = 763)
EGFR_3'UTRc	A (C)	0.163	A/A (n = 761	A/C (n = 301)	C/C (n = 27)
EGFR_in2a	(C) T	0.097	C/C (n = 8)	C/T (n = 185)	T/T (n = 840)
EGFR_in2b	C (T)	0.099	C/C (n = 843)	C/T (n = 188)	T/T (n = 9)
FDX1_3'UTR	(C) T	0.056	C/C (n = 1)	C/T (n = 109)	T/T (n = 884)
HBS1L_1994(ex17)	(A) G	0.235	A/A (n = 51)	A/G (n = 399)	G/G (n = 616)
HPGD_3'UTR	G (T)	0.154	G/G (n = 756)	G/T (n = 284)	T/T (n = 21)
HSD11B1_793(ex6)	C (T)	0.061	C/C (n = 942)	C/T (n = 114)	T/T (n = 8)
HSD17B1_3'UTRa	A (C)	0.157	A/A (n = 751)	A/C (n = 300)	C/C (n = 18)
HSD17B4_in18d	(A) T	0.273	A/A (n = 83)	A/T (n = 412)	T/T (n = 565)
HSD17B7_622(ex6)	(A) G	0.374	A/A (n = 137)	A/G (n = 498)	G/G (n = 398)
HYAL1_83(ex1)	C (T)	0.336	C/C (n = 464)	C/T (n = 470)	T/T (n = 118)
IQGAP2_3'UTR	A (G)	0.231	A/A (n = 606)	A/G (n = 389)	G/G (n = 46)
MIS_in1	(C) T	0.05	C/C (n = 3)	C/T (n = 96)	T/T (n = 929)
MMP1_279(ex2)	(A) G	0.237	A/A (n = 58)	A/G (n = 374)	G/G (n = 603)
NGFIB_in3	C (T)	0.298	C/C (n = 543)	C/T (n = 428)	T/T (n = 108)
NGFIB_1195(ex4)	(A) G	0.243	A/A (n = 53)	A/G (n = 401)	G/G (n = 590)
NGFIB_in4	(A) G	0.245	A/A (n = 59)	A/G (n = 397)	G/G (n = 596)
NGFIB_1374(ex5)	(C) T	0.249	C/C (n = 60)	C/T (n = 421)	T/T (n = 605)
PAPSS2_3'UTRb	(A) G	0.231	A/A (n = 57)	A/G (n = 387)	G/G (n = 639)
PPP1R1A_291(ex5)	(A) G	0.022	-	A/G (n = 48)	G/G (n = 1043)
PRKAB2_3'UTRa	(A) T	0.203	A/A (n = 41)	A/T (n = 342)	T/T (n = 663)
RNF14_3'UTR	C (G)	0.468	C/C (n = 300)	C/G (n = 495)	G/G (n = 234)
SARG_3'UTR	(A) G	0.256	A/A (n = 70)	A/G (n = 395)	G/G (n = 580)
SOX9_in2c	(C) G	0.266	C/C (n = 76)	C/G (n = 386)	G/G (n = 550)
SRD5A2_3'UTRd	(C) T	0.223	C/C (n = 54)	C/T (n = 353)	T/T (n = 627)
STARD3_3'UTR	C (T)	0.036	C/C (n = 983)	C/T (n = 74)	T/T (n = 1)
TSPYL4_3'UTR	C (G)	0.373	C/C (n = 397)	C/G (n = 508)	G/G (n = 134)
UGT1A1_325(ex1)	(C) T	0.081	C/C (n = 7)	C/T (n = 156)	T/T (n = 886)
UGT1A1_in3a	(A) G	0.218	A/A (n = 54)	A/G (n = 370)	G/G (n = 670)
UGT1A1_in3b	(C) T	0.141	C/C (n = 22)	C/T (n = 252)	T/T (n = 778)
UGT1A10_3'UTR	A (G)	0.079	A/A (n = 894)	A/G (n = 155)	G/G (n = 6)
URB_2730(ex7)	A (G)	0.16	A/A (n = 722)	A/G (n = 296)	G/G (n = 18)

SNP ID indicates gene name and basepair from **A**TG start codon, exon number is shown between brackets and if the SNP is in an intron, the intron number is indicated. MAF is the estimated minor allele frequency, and the minor allele is indicated between brackets. Number of animals (n) is shown between brackets after each genotype. DEL = deletion.

**Table 3:** SNPs used for the final association analyses in Norwegian Landrace.

**SNP ID**	**Alleles**	**MAF**	**Homozygote 1**	**Heterorozygote**	**Homozygote 2**
AK1_483(ex5)	(C) T	0.263	C/C (n = 109)	C/T (n = 677)	T/T (n = 914)
AKR1C3_in4b	C (T)	0.37	C/C (n = 1349)	C/T (n = 285)	T/T (n = 20)
AKR1C3_in4c	C (T)	0.095	C/C (n = 1324)	C/T (n = 268)	T/T (n = 19)
ALB_1103(ex9)	(C) T	0.093	C/C (n = 10)	C/T (n = 293)	T/T (n = 1374)
Bap1_3'UTRa	(A) T	0.415	A/A (n = 266)	A/T (n = 851)	T/T (n = 548)
Bap1_3'UTRb	(A) G	0.336	A/A (n = 171)	A/G (n = 751)	G/G (n = 705)
CRSP9_504(ex1)	(A) G	0.106	A/A (n = 17)	A/G (n = 320)	G/G (n = 1333)
CTNND1_3'UTRb	A (G)	0.449	A/A (n = 512)	A/G (n = 816)	G/G (n = 342)
CYB5_-8(prom)	G (T)	0.027	G/G (n = 1611)	G/T (n = 92)	-
CYP11B1_in1c	C (T)	0.492	C/C (n = 435)	C/T (n = 783)	T/T (n = 409)
CYP11B1_in1a	(C) G	0.49	C/C (n = 398)	C/G (n = 842)	G/G (n = 432)
CYP21_in6a	C (G)	0.241	C/C (n = 946)	C/G (n = 643)	G/G (n = 81)
CYP21_in9	A (G)	0.231	A/A (n = 986)	A/G (n = 608)	G/G (n = 83)
CYP21_in8b	(A) G	0.183	A/A (n = 50)	A/G (n = 518)	G/G (n = 1124)
CYP2C49_1251(ex8)	(A) C	0.488	-	A/C (n = 1560)	C/C (n = 39)
CYP2D6_1276(ex7)	C (T)	0.222	C/C (n = 1003)	C/T (n = 604)	T/T (n = 71)
CYP2D6_1287(ex7)	(A) G	0.197	A/A (n = 21)	A/G (n = 594)	G/G (n = 1002)
CYP2E1_1422(ex9)	(C) T	0.417	C/C (n = 286)	C/T (n = 829)	T/T (n = 565)
CYP2E1_1423(ex9)	(A) G	0.183	A/A (n = 55)	A/G (n = 493)	G/G (n = 1103)
CYP2E1_in1a	C (G)	0.416	C/C (n = 562)	C/G (n = 798)	G/G (n = 285)
CYP2E1_in1b	C (DEL)	0.189	C/C (n = 1086)	C/D (n = 519)	D/D (n = 55)
CYP2E1_in6	C (T)	0.186	C/C (n = 1064)	C/T (n = 497)	T/T (n = 52)
CYP3A4_3'UTR	(C) T	0.335	C/C (n = 168)	C/T (n = 804)	T/T (n = 728)
CYP3A4_1498(ex13)	(A) G	0.331	A/A (n = 163)	A/G (n = 786)	G/G (n = 729)
DHRS6_3'UTR	(C) T	0.333	C/C (n = 192)	C/T (n = 720)	T/T (n = 748)
EGFR_3'UTRa	C (T)	0.447	C/C (n = 482)	C/T (n = 795)	T/T (n = 313)
EGFR_3'UTRb	C (T)	0.439	C/C (n = 493)	C/T (n = 817)	T/T (n = 296)
EGFR_3'UTRc	A (C)	0.131	A/A (n = 1262)	A/G (n = 390)	G/G (n = 24)
EGFR_in12	C (T)	0.449	C/C (n = 503)	C/T (n = 858)	T/T (n = 332)
EGFR_in2a	(C) T	0.132	C/C (n = 26)	C/T (n = 384)	T/T (n = 1236)
EGFR_in2b	(C) T	0.417	C/C (n = 298)	C/T (n = 809)	T/T (n = 578)
HBLD2_3'UTRa	G (T)	0.488	G/G (n = 409)	G/T (n = 783)	T/T (n = 371)
HSD11B1_793(ex6)	(C) T	0.306	C/C (n = 155)	C/T (n = 710)	T/T (n = 804)
HSD17B1_3'UTRb	(G) T	0.101	G/G (n = 20)	G/T (n = 293)	T/T (n = 1335)
HSD17B1_3'UTRa	(A) C	0.13	A/A (n = 19)	A/C (n = 397)	C/C (n = 1256)
HSD17B7_622(ex6)	A (G)	0.276	A/A (n = 854)	A/G (n = 593)	G/G (n = 141)
HSPCA_3'UTR	C (T)	0.429	C/C (n = 527)	C/T (n = 811)	T/T (n = 294)
HSPCA_2175(ex9)	A (G)	0.433	A/A (n = 538)	A/G (n = 833)	G/G (n = 311)
HYAL1_748(ex1)	(G) T	0.488	G/G (n = 429)	G/T (n = 872)	T/T (n = 390)
HYAL1_75(ex1)	(C) T	0.489	C/C (n = 390)	C/T (n = 863)	T/T (n = 427)
HYAL2_583(ex1)	(A) C	0.486	A/A (n = 380)	A/C (n = 819)	C/C (n = 424)
HYAL2_in1b	A (G)	0.488	A/A (n = 428)	A/G (n = 874)	G/G (n = 389)
IQGAP2_3'UTR	A (G)	0.27	A/A (n = 837)	A/G (n = 640)	G/G (n = 109)
MIS_in1	C (T)	0.13	C/C (n = 1288)	C/T (n = 383)	T/T (n = 30)
MMP1_in3a	C (T)	0.253	C/C (n = 927)	C/T (n = 633)	T/T (n = 104)
MMP13_in2	(A) C	0.269	A/A (n = 113)	A/C (n = 667)	C/C (n = 881)
NGFIB_in3	C (T)	0.052	C/C (n = 1509)	C/T (n = 167)	T/T (n = 3)
PAPSS2_3'UTRa	A (G)	0.269	A/A (n = 861)	A/G (n = 620)	G/G (n = 121)
PIAS1_1863(ex14)	A (G)	0.266	A/A (n = 885)	A/G (n = 679)	G/G (n = 104)
PPP1R1A_291(ex5)	(A) G	0.103	A/A (n = 16)	A/G (n = 314)	G/G (n = 1343)
RALBP1_3'UTR	C (T)	0.025	C/C (n = 1585)	C/T (n = 81)	T/T (n = 2)
RNF14_3'UTR	(C) G	0.433	C/C (n = 297)	C/G (n = 843)	G/G (n = 520)
SARG_3'UTR	A (G)	0.192	A/A (n = 1113)	A/G (n = 508)	G/G (n = 71)
SOX9_in2c	C (G)	0.406	C/C (n = 588)	C/G (n = 750)	G/G (n = 284)
SRD5A2_3'UTRd	C (T)	0.478	C/C (n = 449)	C/T (n = 826)	T/T (n = 377)
SRD5A2_3'UTRa	G (T)	0.176	G/G (n = 1122)	G/T (n = 506)	T/T (n = 41)
STARD3_3'UTR	C (T)	0.133	C/C (n = 1279)	C/T (n = 384)	T/T (n = 33)
TSPYL4_3'UTR	C (G)	0.408	C/C (n = 588)	C/G (n = 830)	G/G (n = 275)
UGT1A1_325(ex1)	(C) T	0.105	C/C (n = 19)	C/T (n = 301)	T/T (n = 1296)
UGT1A10_3'UTR	(A) G	0.115	A/A (n = 66)	A/G (n = 241)	G/G (n = 1310)
URB_2730(ex7)	A (G)	0.166	A/A (n = 1139)	A/G (n = 464)	G/G (n = 40)

SNP ID indicates gene name and basepair from **A**TG start codon, exon number is shown between brackets and if the SNP is in an intron, the intron number is indicated. MAF is the estimated minor allele frequency, and the minor allele is indicated between brackets. Number of animals (n) is shown between brackets after each genotype. DEL = deletion.

**Table 4:** Descriptive statistics for phenotypes in Duroc.

Phenotype	Breed	n	Mean	SD	Min	Max
Bulbo Urethralis length (cm)	D	469	11.55	1.67	7.5	19.75
Skatole (ppm)	D	934	0.06	0.11	0.00	1.51
ln(Skatole)	D	934	-4.10	2.25	-9.21	0.41
Indole (ppm)	D	934	0.04	0.05	0.00	0.61
ln(Indole)	D	934	-3.63	0.77	-9.21	-0.50
Androstenone fat (ppm)	D	950	3.27	2.79	0.01	20.5
ln(Androstenone fat)	D	950	0.84	0.88	-4.60	3.02
Androstenone plasma (ppm)	D	786	20.1	15.6	0.03	95.2
ln(Androstenone plasma)	D	785	2.70	0.87	-3.5	4.56
Testosterone (ppm)	D	934	12.9	9.85	0.00	107
ln(Testosterone)	D	933	2.17	1.44	-9.21	4.67
Estrone sulphate (ppm)	D	934	28.8	20.3	0.00	148
ln(Estrone sulphate)	D	933	3.05	1.01	-9.21	5.00
17β-estradiol (ppm)	D	935	0.24	0.14	0.03	1.10
ln(17β-estradiol)	D	934	-1.57	0.52	-3.61	0.10

Mean, standard deviation (SD), minimum (min) and maximum (max) values are presented for all the phenotypes included in the association study. Number of animals (n) used in the SNP analyses for each trait is indicated. For all phenotypes except bulbo urethralis length, the data were ln-transformed to get a normal distribution and both original and ln-transformed values are presented.

**Table 5:** Descriptive statistics for phenotypes in Norwegian Landrace.

Phenotype	Breed	n	Mean	SD	Min	Max
Bulbo Urethralis length (cm)	NL	757	10.98	1.45	7.25	16.5
Skatole (ppm)	NL	1488	0.10	0.15	0.00	1.87
ln(Skatole)	NL	1488	-2.93	1.22	-9.21	0.63
Indole (ppm)	NL	1488	0.04	0.07	0.00	1.19
ln(Indole)	NL	1488	-3.51	0.76	-6.81	0.17
Androstenone fat (ppm)	NL	1525	1.16	1.14	0.04	13.4
ln(Androstenone fat)	NL	1525	-0.18	0.79	-3.22	2.60
Androstenone plasma (ppm)	NL	1371	11.0	8.29	0.47	112
ln(Androstenone plasma)	NL	1371	2.18	0.69	-0.75	4.72
Testosterone (ppm)	NL	1509	7.04	6.79	0.00	161
ln(Testosterone)	NL	1509	1.44	1.71	-9.21	5.08
Estrone sulphate (ppm)	NL	1509	13.4	12.6	0.00	205
ln(Estrone sulphate)	NL	1509	2.21	1.00	-9.21	5.32
17β-estradiol (ppm)	NL	1509	0.14	0.08	0.03	1.67
ln(17β-estradiol)	NL	1509	-2.04	0.43	-3.68	0.51

Mean, standard deviation (SD), minimum (min) and maximum (max) values are presented for all the phenotypes included in the association study. Number of animals (n) used in the SNP analyses for each trait is indicated. For all phenotypes except Bulbo Urethralis length, the data were ln-transformed to get a normal distribution and both original and ln-transformed values are presented.

**Table 6:** Significant results for SNP associations in Duroc and Norwegian Landrace.

Trait	SNP	LRT D	LRT NL	% of tot.var. D	% of tot.var. NL	Favourable allele
ln(Androstenone fat)	NGFIB_in4	5.99		5.1		A
	CTNND1_3'UTRa	6.02		16.3		T
	CYP2D6_1276(ex7)	6.35		5.6		C
	CYP2C49_1083(ex7)	7.33		12.3		C
						
ln(Androstenone plasma)	BAP1_3'UTRb		5.40		1.3	A
	HYAL2_583(ex1)		5.53		1.6	A
	BAP1_3'UTRa		6.45		2.0	A
	SRD5A2_3'UTRd		8.80		2.1	T
						
ln(Indole)	CYP21_in9	8.52		6.3		A
	CYP21_in6b	9.36		7.3		C
	CYP21_in8b	10.4		6.4		G
	CYP2E1_1422(ex9)		28.3		4.5	C
	CYP2E1_1423(ex9)	25.4	39.5	6.5	4.9	A
	CYP2E1_in1a	11.9	19.6	7.5	3.1	G
	CYP2E1_in1b	24.8	35.4	7.0	4.8	DEL
	CYP2E1_in6	26.4	31.8	7.4	4.5	A
						
ln(Skatole)	CYP21_in9	7.86		12.4		A
	CYP21_in6b	7.20		13.4		C
	CYP2E1_1423(ex9)	6.81	12.0	2.5	3.0	A
	CYP2E1_in1b	8.38	8.80	3.4	2.1	DEL
	CYP2E1_in6	7.79	9.10	3.1	2.2	A
						
ln(Estrone sulphate)	AKR1C3_in4b	5.92		1.5		T
	AKR1C3_in4c	6.57		1.9		T
	AK1_483(ex5)	13.8		16.2		T
	SRD5A2_3'UTRd		6.30		1.3	C
						
ln(17β-estradiol)	AKR1C3_in4c	5.60		2.0		T
	PPP1R1A_291(ex5)		5.71		1.3	G
						
ln(Testosterone)	HYAL2_583(ex1)		6.28		1.5	C
	BAP1_3'UTRa		6.80		1.8	T

Significance level is presented (LRT) together with percentage of the total phenotypic variance explained by the SNP and the favourable allele. DEL = deletion.

In Duroc, 4 SNPs were associated with levels of androstenone in adipose tissue and they were found to explain 5.1% (*NGFIB_in4*), 5.6% (*CYP2D6_1276(ex7)*), 12.3% (*CYP2C49_1083(ex7)*) and 16.3% (*CTNND1_3'UTRa*) of the total variation. In NL, none of the SNPs examined in this study was significantly associated with levels of androstenone in adipose tissue, but 4 SNPs were significantly associated with levels of androstenone in plasma. These SNPs explain 1.3% (*BAP1_3'UTRb*), 1.6% (*HYAL2_583(ex1)*), 2% (*BAP1_3'UTRa*) and 2.1% (*SRD5A2_3'UTRd*) of the total variation. Levels of skatole in adipose tissue were significantly associated with 5 SNPs within two different genes in D and 3 SNPs from one gene in NL. In D, SNPs within *CYP21 *explained 12.4% and 13.4% of the total variation while SNPs within *CYP2E1 *explained between 2.5% and 3.1% of the total variation. *CYP2E1 *SNPs in NL explained between 2.1% and 3% of the total skatole variation. Association results for levels of indole in adipose tissue show that 7 SNPs from two genes (*CYP21 *and *CYP2E1*) explain between 6.3% and 7.5% of the total variation in D and that 5 SNPs from *CYP2E1 *explain between 3.1% and 4.9% of the total variation in NL. Some SNPs were also associated with levels of other steroids but none of them were associated with the length of bulbo urethral gland (Table 6).

**Figure 1:**
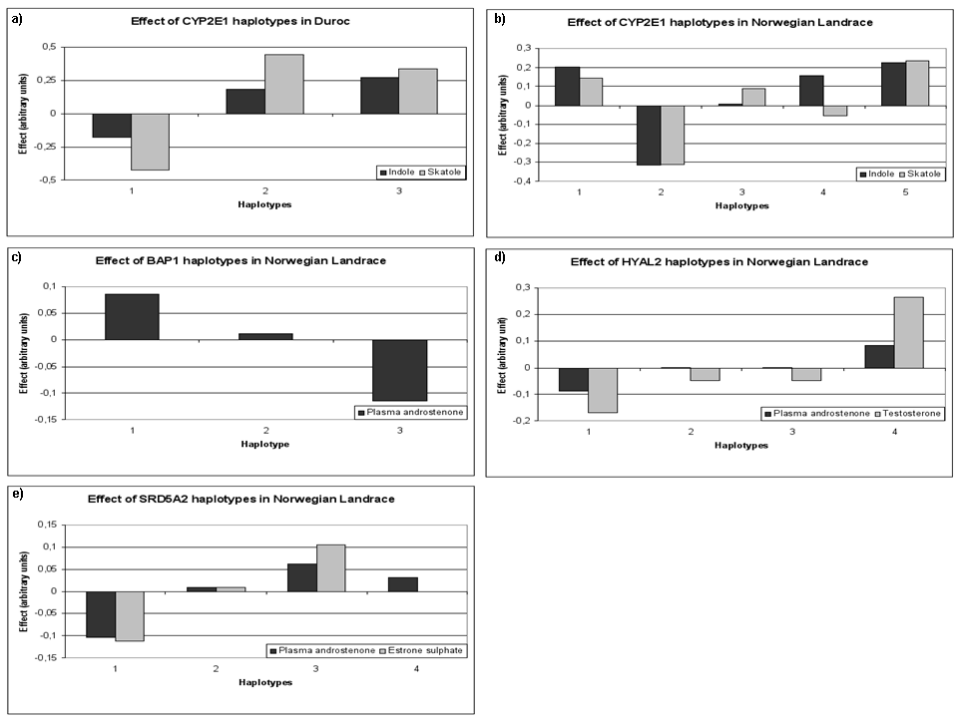
**Significant effects of haplotypes**. Significant effects were found for *CYP2E1 *haplotypes in Duroc (a) and Norwegian Landrace (b), *BAP1 *haplotypes (c), *HYAL2 *haplotypes (d) and *SRD5A2 *haplotypes (e) in Norwegian Landrace.

Haplotypes were constructed for genes with more than one SNP and an additional association analysis was performed on the phenotypes found to be significant in the single SNP analysis. This resulted in 5 significant genes for D (Table 7) and 5 for NL (Table 8). Only haplotypes with frequencies of more than 1% were included in the statistical analyses. Significant haplotype effects on the phenotypes investigated are shown in Table 9. The haplotype analyses of *CYP2E1 *were significant in both breeds for levels of skatole and indole and analyses suggest that as much as 12% of the total variation in indole and 6% of the total variation in skatole can be explained by this gene in D. In NL, 9.5% and 4.6% of the total variation in indole and skatole, respectively, was explained by *CYP2E1 *haplotypes. The effect of different *CYP2E1 *haplotypes is presented in Figure 1a for D and in Figure 1b for NL. Significant effects were also found for *BAP1*, *HYAL2 *and *SRD5A2 *and levels of androstenone in Norwegian Landrace and these haplotypes are presented in Figure 1c, d and 1e, respectively.

**Table 7:** Haplotypes and their frequencies in Duroc.

Gene	Haplotype no.	Haplotype	Frequency
CYP21	1	TATCG	0.27
	2	TACCG	0.36
	3	TACAG	0.30
	4	CGCCA	0.06
	5	CGCAA	0.01
			
CYP2D6	1	TG	0.08
	2	TA	0.15
	3	CG	0.77
			
CYP2E1	1	GDTT	0.47
	2	GCCC	0.22
	3	CCCC	0.29
			
CTNND1	1	TG	0.89
	2	GA	0.11
			
NGFIB	1	CAAC	0.24
	2	CGGT	0.45
	3	TGGT	0.30

Haplotypes with frequencies less than 1% were excluded from analyses. D = deletion.

**Table 8:** Haplotypes and their frequencies in Norwegian Landrace.

Gene	Haplotype no.	Haplotype	Frequency
BAP1	1	TG	0.58
	2	AG	0.08
	3	AA	0.33
			
CYP2D6	1	CG	0.76
	2	CA	0.02
	3	TG	0.03
	4	TA	0.19
			
CYP2E1	1	CCCAC	0.55
	2	GDTGT	0.16
	3	GCCGC	0.22
	4	GCCAC	0.02
	5	CCCGC	0.01
			
HYAL2	1	AG	0.47
	2	AA	0.02
	3	CG	0.02
	4	CA	0.5
			
SRD5A2	1	GT	0.47
	2	TT	0.01
	3	GC	0.36
	4	TC	0.16

Haplotypes with frequencies less than 1% were excluded from analyses. D = deletion.

**Table 9:** Significant results for haplotype associations.

Trait	Breed	Sign. haplotype	LRT	% of tot.var.	Favourable haplotype
ln(Indole)	D	CYP2E1	21.6	12.2	GDTT
ln(Skatole)	D	CYP2E1	8.81	6.1	GDTT
ln(Androstenone plasma)	NL	SRD5A2	10.5	2,0	GT
ln(Androstenone plasma)	NL	BAP1	6.71	2.7	AA
ln(Androstenone plasma)	NL	HYAL2	6.23	2.6	AG
ln(Testosterone)	NL	HYAL2	5.68	2.5	CA
ln(Estrone sulphate)	NL	SRD5A2	8.92	1.5	GC
ln(Indole)	NL	CYP2E1	35.7	9.5	GDTGT
ln(Skatole)	NL	CYP2E1	8.67	4.6	GDTGT

Significance level is presented (LRT) together with percentage of the total phenotypic variance explained by the haplotype and the favourable allele. D = deletion.

## Discussion

Unfavourable correlations between boar taint compounds and phenotypes related to reproduction make selection against high levels of boar taint challenging. Analysing the effect of candidate genes on androstenone, skatole and sex steroids will give new information about which genes that affect different compounds. It may also reveal polymorphisms that can be used for breeding against high levels of boar taint without simultaneously affecting fertility and reproduction in the pigs. In this study we report a larger set of SNPs in selected genes involved in biosynthesis and metabolism of androstenone and skatole, in addition to regulatory factors likely involved in boar taint. The SNPs were genotyped in boars from Duroc (D) and Norwegian Landrace (NL) and tested for associations to 8 different phenotypes related to boar taint and reproduction.

One of the key enzymes in the metabolism of skatole is cytochrome P450 family member CYP2E1 [15,50]. A total of 7 metabolites have been identified for skatole [51] and 5 of them were produced at decreased levels when a CYP2E1 inhibitor was present [52]. Moreover, levels of CYP2E1 mRNA [53], protein [15,53], and enzyme activity [54] have been found negatively associated with levels of skatole in adipose tissue of boars. It has also been shown that CYP2E1 is induced by skatole [55] and inhibited by androstenone and 17β-estradiol [56]. In our study we found no association of CYP2E1 and androstenone. This is in accordance with the results of Zamaratskaia *et al*. [57]. We did, however, find significant associations between SNPs and haplotypes within *CYP2E1 *and levels of skatole and indole in both breeds. These results are in agreement with associations previously found between *CYP2E1_1423(ex9) *and levels of skatole [58], although in another study there was no significant associations detected in Large White × Meishan crossbreds [59]. Both SNP and haplotype analyses showed that the extent of variation in skatole and indole explained by *CYP2E1 *is higher in D than NL. Breed specific differences for CYP2E1 is in agreement with Doran *et al*. [53]. In both breeds we identified a haplotype that was associated with reduced levels of both skatole and indole, and this haplotype contains the favourable alleles from the SNP analysis.

Cytochrome P450 family member CYP21 is involved in the steroid biosynthesis pathway [60]. It has previously been suggested as a positional candidate gene for androstenone [61]. We identified 7 SNPs in the introns of this gene. None of these SNPs were found to be associated with levels of any of the steroids included in this study. On the contrary, 3 SNPs were significantly associated with levels of skatole and indole in D, but not in NL. Two haplotypes containing all the favourable alleles have an advantageous effect on levels of indole and skatole in D. This effect was, however, not significant (LRT of 3.4 (p < 0.01) for indole and 1.6 (p < 0.1) for skatole). The role of CYP21 regarding to levels of skatole and indole needs to be clarified.

Cytochrome P450 porcine isoform CYP2C49 is a member of the subfamily CYP2C, which metabolise drugs and steroids [62]. We previously identified this isoform as the most significant gene in a gene expression study including pigs with extreme high and low levels of androstenone [37] and in the current study we identified 4 SNPs in the *CYP2C49 *gene. A significant association was shown between *CYP2C49_1083(ex7) *and levels of androstenone in adipose tissue in D, and this SNP explain 12.3% of the total variation. This result suggests that the level of gene expression is regulated within the gene. The SNP was not significantly associated with any of the other phenotypes in the study. A microsatellite linked to *CYP2C18*, which is the human ortholog of *CYP2C49*, has previously been tested for association with levels of skatole in adipose tissue, in accordance with our results, no significant results were obtained [63].

Cytochrome P450 member CYP2D6 is involved in drug metabolism and has a broad substrate specificity [64]. Some studies have also suggested that CYP2D6 is regulated by steroid hormones [65,66]. In this study we found significant association between the SNP *CYP2D6_1276(ex7) *and level of androstenone in adipose tissue of D boars. SNPs in *CYP2D6 *showed no significant associations to levels of skatole or indole. This result is supported by the study of Diaz and Squires [52], showing that inhibition of CYP2D6 do not to affect production of skatole metabolites. Moreover, we found no associations with levels of testosterone or estrogens.

Catenin delta, *CTNND1*, is part of the catenin family that functions in intracellular signalling and transcriptional regulation [67] and phosporylation of CTNND1 leads to regulation of several transcription factors [67]. A SNP in *CTNND1 *was significantly associated with levels of androstenone in adipose tissue of D boars. No associations were found for any of the other phenotypes analysed, which makes *CTNND1 *interesting for selection against high levels of androstenone in D. Such regulators, however, will likely affect several other biological processes and this need to be considered before implementation in the selection scheme.

Orphan nuclear receptor family member NGFI-B, also known as Nurr77, is involved in transcriptional regulation of several steroidogenic genes, including steroidogenic acute regulatory protein (*StAR*), 3β-hydroxysteroid dehydrogenase (*3β-HSD*), cytochrome P450 c17 (*CYP17*), and *CYP21 *[68,69]. A SNP in *NGFI-B *was significantly associated with levels of androstenone in adipose tissue of D boars. It was not associated with any of the other phenotypes examined and is therefore also interesting as a possible genetic marker for selection against androstenone.

Short-chain dehydrogenase/reductase family members SRD5A1 and SRD5A2 are enzymes that catabolise a number of steroids into their 5α-reduced metabolites [70]. SRD5A2 also catalyses the final step of androstenone formation [71]. No relationship was found between genetic variation of SRD5A2 and androstenone in a Large White × Meishan crossbreed [61]. In our study, however, 6 SNPs were detected in the 3' UTR region of porcine *SRD5A2 *and the SNP *SRD5A2_3'UTRd *was significantly associated with levels of androstenone and estrone sulphate in plasma of NL boars. The haplotype analyses confirmed these associations. The haplotype associated with reduced levels of androstenone, however, was also associated with reduced levels of estrone sulphate, which makes it less desirable for selection purposes. Aldo-keto reductase AKR1C isoforms work together with SRD5As in liver catabolism of steroids [72] and we previously found the isoform *AKR1C4 *differentially expressed regarding to levels of androstenone in pig testes [36]. SNPs in isoform *AKR1C3 *were associated with levels of both estrone sulphate and estradiol in D boars, but no association with levels of androstenone suggest different functions for the two isoforms *AKR1C3 *and *AKR1C4*.

The breast/ovarian cancer susceptibility associated protein-1 (BAP1) has been implied to alter substrate function through post-translational modifications [73]. It has the same structure and functions as the ubiquitin carboxy-terminal hydrolase (UCH) family, which are involved in ubiquitin-mediated regulatory pathways [73]. Two SNPs in *BAP1 *were significantly associated with levels of androstenone in plasma of NL boars. Moreover, a *BAP1 *haplotye was associated with decreased levels of androstenone. One of the SNPs in BAP1 was, however, also associated with levels of testosterone in plasma in the same breed. The favourable allele with respect to androstenone was also associated with reduced levels of testosterone, which suggest that this SNP is not appropriate for selection purposes.

Hyaluronoglucosaminidases are enzymes responsible for hyaluronan metabolism. It has been shown that hyaluronan metabolic products can induce expression of heat-shock proteins [74], which are known to activate for example steroid hormone receptors [75]. Metabolism of hyaluronan has also been shown to change during testicular development and is associated with testicular descent [76]. We have identified SNPs in *HYAL1, HYAL2 *and *HYAL3*. Among these a SNP in *HAYL2 *was associated with levels of androstenone in plasma of NL boars, and a haplotype with negative effect on the levels of androstenone was also identified. This *HYAL2 *SNP and the haplotype were also associated with levels of testosterone in NL. The favourable haplotype for levels of androstenone is also associated with reduced levels of testosterone, implying that it is not so suitable for breeding purposes.

Except from the associations found for SNPs in *CYP2E1 *with levels of skatole and indole in adipose tissue, our results show breed differences with respect to significantly associated SNPs. The breed differences are in agreement with results from our previous expression profiling for D and NL breeds [36,37,77]. Different associations suggest that D and NL have different linkage disequilibrium (LD) with the causative mutation. It is therefore likely that there is some distance between the SNP and the causative mutation. Moreover, fixation or nearly fixation was observed for many candidate gene SNPs and different SNPs were monomorphic in D and NL. It emphasises the importance of testing polymorphisms in the population in question before using them for breeding purposes. When including all the significant SNPs for a trait in the same model there were some problems of convergence, which might indicate that the SNP effects are correlated and partly explain the same variation. Although a rather strict significance threshold corresponding to p < 0.001 was used in this study, the large number of tests performed suggests that there might still be false positive results and the findings should be confirmed in another study. Moreover, SNPs that are significant for boar taint compounds and not for phenotypes related to reproduction might still have a small effect on reproduction even though this study was not able to detect it.

## Conclusion

This study reports significant associations between SNPs within *CYP21 *and *CYP2E1 *and reduced levels of both skatole and indole. Moreover, a number of SNPs within *CYP2C49, CYP2D6*, *NGFIB *and *CTNND1 *were found significantly associated with levels of androstenone in adipose tissue. These SNPs did not reveal obvious associations with levels of testosterone or estrogens, which might indicate that they can be implemented in practical breeding to reduce levels of androstenone and skatole without causing simultaneous negative consequences on phenotypes related to reproduction.

## Abbreviations

3β-HSD: 3β-hydroxysteroid dehydrogenase; AKR1C3: aldo-keto reductase family member 1C3; AKR1C4: aldo-keto reductase family member 1C4; AOX: aldehyde oxidase; BAP1: BRCA1 associated protein; CTNND1: catenin delta; CYB5: cytochrome b5; CYP17: cytochrome P450 family member 17; CYP21: cytochrome P450 family member 21; CYP2C49: cytochrome P450 family member 2C49; CYP2D6: cytochrome P450 family member 2D6; CYP2E1: cytochrome P450 family member 2E1; DHRS8: short-chain dehydrogenase/reductase member 8; D: Duroc; FDX1: ferredoxin; FTH1: ferritin heavy polypeptide; HYAL1: hyaluronoglucosaminidase 1; HYAL2: hyaluronoglucosaminidase 2; HYAL3: hyaluronoglucosaminidase 3; LD: linkage disequilibrium; MAF: minor allele frequency; NGFIB: orphan nuclear receptor member NGFI-B; NL: Norwegian Landrace; SNP: single nucleotide polymorphism; SRD5A2: steroid 5α-reductase; StAR: steroidogenic acute regulatory protein; UCH: ubiquitin carboxy-terminal hydrolase.

## Authors' contributions

MM conducted SNP molecular work, was involved in statistical analyses and drafted the paper. SL was involved in planning the project, provided laboratory facilities and took part in writing the paper. TA carried out statistical analyses. THEM was involved in statistical supervision. MHSH conducted SNP molecular work. CB provided SNPs from the Sino-Danish sequencing project. EG coordinated the study, was involved in planning the project, performed statistical analyses and took part in writing the paper. All authors have read and approved the final manuscript.

## Supplementary Material

Additional file 1 **Genotyped SNPs**. Genotyped SNPs are presented with gene name and accession number. Position indicates base pairs from **A**TG start codon according to human reference sequence. Exon number is presented between brackets and if the SNP is in an intron, the intron number is indicated. An asterix is designated to SNP alleles causing amino acid change. Step 1+2 indicates the SNPs that were genotyped in all animals whereas step 1 indicates the SNPs that were initially genotyped in 380 animals per breed and discarded due to low significance levels (NS) or failing assays (-). Click here for file

Additional file 2 **Primer sequences for genotyping**. Forward, reverse and extension primers for genotyping on the Sequenom MassARRAY system (Sequenom, San Diego, USA) are presented. The multiplexes are indicated in the first column. Multiplexes W1-W6 represents primers for the SNPs that were genotyped in all animals whereas 1 indicates primers for the SNPs that were initially genotyped in the first 760 animals and discarded. Click here for file
