# Supplementary material for: Association between SNPs within candidate genes and compounds related to boar taint and reproduction
Source: BMC Genet. 2009 Jul 5;10:32. doi: 10.1186/1471-2156-10-32 (PMC2723134; doi:10.1186/1471-2156-10-32)
Supplement: Additional file 2 — Primer sequences for genotyping. Forward, reverse and extension primers for genotyping on the Sequenom MassARRAY system (Sequenom, San Diego, USA) are presented. The multiplexes are indicated in the first column. Multiplexes W1-W6 represents primers for the SNPs that were genotyped in all animals whereas 1 indicates primers for the SNPs that were initially genotyped in the first 760 animals and discarded. [file 1471-2156-10-32-S2.doc]

| Multiplex | Oligo name | Oligo sequence (forward) | Oligo sequence (reverse) | Extension primer |
| --- | --- | --- | --- | --- |
| W1 | HYAL2_in1b | ACG TTG GAT GTC TCA CCA ATG GTG GAG ATG | ACG TTG GAT GTC TTC CCC AGT GAC CAT CTC | CCA TCT CCT TCC CAC |
| W1 | HSD17B4_in18d | ACG TTG GAT GCT CAT GGA GTT CCT GTA GTG | ACG TTG GAT GTC CTT ACC CCA CTA TGC AAG | GAA CCC ACA ACC TCA |
| W1 | HYAL1_75(ex1) | ACG TTG GAT GTT GCA TTC CAG ATG GTG GTG | ACG TTG GAT GTC CTG AAC TTG CTC AGC GTG | AGG ATC CAG GGA CCC |
| W1 | CYP21_in8a | ACG TTG GAT GAG TTA GAG GCC ACC AGG AC | ACG TTG GAT GTT GCC ACA CCG AGC CAC TC | GCC TAG CAG GTG ACT C |
| W1 | CYP11B1_in1c | ACG TTG GAT GGT CAA ATC TGT GGC ATG GAG | ACG TTG GAT GTC GCA CTG GTT CTG CAG AGT | GGC CAG GGG AGG ACA C |
| W1 | HSD17B1_3'UTRb | ACG TTG GAT GAG TGG TCC TCG ATC CCC AAG | ACG TTG GAT GAA GCC AAG TCA CAA GCC AAC | GCG CCA ACT CTC CCC GC |
| W1 | Hsp70_939(ex1) | ACG TTG GAT GAG CTG TGC TCG GAC CTG TTC | ACG TTG GAT GAT CTG GGC CTT GTC CAG CTT | TTC TCC ACC GGC TCC AG |
| W1 | STARD13_3'UTR | ACG TTG GAT GGC TAA TCA CAT CAC TCC TGC | ACG TTG GAT GTT GAC AAA CAA GCT CGA TGC | AAG CTC GAT GCA TCT TT |
| W1 | CYB5_-8(prom) | ACG TTG GAT GCT CTG TTC CGC TCA TCT CTG | ACG TTG GAT GAT ACT TCA CGG CTT TGT CGG | CTT TCG GCC ATT TCG TAA |
| W1 | ATP5F1_183(ex3) | ACG TTG GAT GCT GTC ACT CCA GTT TTA GG | ACG TTG GAT GGG AGG AAA AGT TCG TTT GGG | TTT GGG GCT GAT CCC TGA |
| W1 | HSD17B4_in18a | ACG TTG GAT GCT GTT TAT GAC TGG TAG TTT G | ACG TTG GAT GCT GAA CAG TAC TTC CTA CCT | ACT TCC TAC CTT TCC ATT C |
| W1 | SRD5A2_3'UTRb | ACG TTG GAT GCC TCT GCA TAA AAG CAG ACC | ACG TTG GAT GAT TCT GCA CGG GAC CCC TTA | CTG GAC CCC TTA GTG TTT C |
| W1 | STARD3_3’UTR | ACG TTG GAT GTT CTT CCT CCC CAG TGT TTG | ACG TTG GAT GTG TTG CCA CAG CTT CCA GAG | GAG CGC CAG AGA GGG CCA G |
| W1 | AK1_483(ex5) | ACG TTG GAT GCG AAC GAT GCC ACG TTT CTC | ACG TTG GAT GAA GAG ACC ATC AAG AAG CGG | CCC GAC ACA GAG CCC GTC AT |
| W1 | AK1_-21(prom) | ACG TTG GAT GCT TCC ATC CTG CCG CGG TC | ACG TTG GAT GGA AGA GGT GGA CAG GAG TG | CCC TTA CAG AGC GCT GAC TC |
| W1 | MMP13_in3b | ACG TTG GAT GTT CCT TCT TTT AGT CAT GC | ACG TTG GAT GAC ACC CAG TGG TAC CTA GTG | TTA CCT AGT GCA GGT ACT TG |
| W1 | AKR1C3_in4d | ACG TTG GAT GTT GTC ATG ACC TCC TTC TCC | ACG TTG GAT GTA AGG GCA GGG AGA AAT AGG | GGG TGG TCA CCT GTC GAG AA |
| W1 | CYP21_in8d | ACG TTG GAT GGA TGA CAA CGG TGC CCT CG | ACG TTG GAT GTC CTG GTG GCC TCT AAC TC | GTG TCC TCT AAC TCC ACC CTT C |
| W1 | PRKAB2_3'UTRa | ACG TTG GAT GGC AGA AAA TAA ACT ATC CCT G | ACG TTG GAT GTG ATC CAG AAG GTA GAG AGG | CTA CAG TAC AGC TCA CTC ATT C |
| W1 | HBLD2_3'UTRb | ACG TTG GAT GAT CAT GTG ACT GTC ACG TGC | ACG TTG GAT GTC GTC TTC AAA ATG CAT CAC | GTT TCC TTG TAA GGC AGT CAT A |
| W1 | PRKAB2_3'UTRb | ACG TTG GAT GGA AGG ATA GGA GTG AGG TTC | ACG TTG GAT GCC CTT CAA ACT CCA ATC GTG | AAC CAT CGT GAT TGG TGC TTA A |
| W1 | SRD5A2_3'UTRa | ACG TTG GAT GGG AAC AAA GTC CCC ACA ATC | ACG TTG GAT GCA CAT ACA CAG TGG GAC TTG | GGG ACT TGA AAA GTT ACA GGT TC |
| W1 | CYP3A4_1498(ex13) | ACG TTG GAT GTT CCT TGA GGA ACC AAG CCC | ACG TTG GAT GTT CTT CTG AAG GTT GTG CCC | AGG GAG TGC CCA GAG ATG GGA CC |
| W1 | PIAS1_1863(ex14) | ACG TTG GAT GTT TGC AAC GGT GTG GCT ATG | ACG TTG GAT GAA TGG AAG CAG TAG TGG CAG | CCT CAT GGT ATC CTC CAA CAG CTT |
| W1 | DECR1_3'UTR | ACG TTG GAT GTC CCT TAA AAG CTG AGA TAG | ACG TTG GAT GGC ATA AAA ATG TGG AGG AGG | AAT TCA TCC TAT CTC TAG AAT TTA |
| W1 | SRD5A2_3'UTRf | ACG TTG GAT GAC AAC ACC CTC TTT TCT CGG | ACG TTG GAT GCA GCA CAT GCT CTT ACT TGG | GAA ACT TTC ATT GTT TCA ATG TAT C |
| W1 | Hsp70_1315(ex1) | ACG TTG GAT GAG GTT GTT GTC CCG CGT CAT | ACG TTG GAT GTC ACC CAC CTA CTC GGA CAA | GCT CCT CGG ACA ACC AGC CGG GCG T |
| W1 | SULT1B1_971(ex7) | ACG TTG GAT GTC ACA GTG GCC CAA AAT GAG | ACG TTG GAT GAG GTT GTT AAA TCT CTG TGC | GGG TTT AAA TCT CTG TGC AGA ATT G |
| W1 | MMP1_177(ex2) | ACG TTG GAT GTG CCA GTG GAA AAG AAA AGG | ACG TTG GAT GTT TCC CCG TTA CTT TCA GCC | CTC CTT TGC TTT AGT TTT TCA ACT AC |
| W1 | HSD17B4_in18c | ACG TTG GAT GCC CGA ATG AAC TGT TCT TGG | ACG TTG GAT GTC CAT GTA AAT GGG CCT ATC | ATT ACA GAG AAT TTG TTA GAT ATA AG |
| W1 | HSD17B7_622(ex6) | ACG TTG GAT GTA TTC CAG TGT GGT GTG TCC | ACG TTG GAT GTT GGC ATG ATC AGC ATC CAC | CCC CAT AAA AGG AGA TAA AAT TCC ATA |
| W1 | HSPCA_2028(ex8) | ACG TTG GAT GAG TCA CCA AAG AGG GCC TG | ACG TTG GAT GCT TCA TGA TCT TGC AGA GG | GCT ACT CCT GTT TCT TCT TCT CCT CTT C |
| W1 | SOX9_in2c | ACG TTG GAT GTG ATT AAC CCG CCA GTA CAC | ACG TTG GAT GAT TTG GTC TTC CGG ACC CTC | CCT CTT CCG GAC CCT CGG GGC CTC AGA C |
| W1 | CYP2E1_in1a | ACG TTG GAT GTA CTG TTG ACC AGG AGA ACC | ACG TTG GAT GCA TAG CAA TGT GCT CCC TAC | CAA GGT GCT CCC TAC GGA ATC CTC AGT G |
| W1 | CYP2E1_1423(ex9) | ACG TTG GAT GTG TTG ACC CCA AGG ATA TCG | ACG TTG GAT GGA CAC AGA GTT TGT AAT GGG | AGG AGG GGA ATC TTG GCA AAC CCA ATC G |
| W2 | UGT1A1_325(ex1) | ACG TTG GAT GCC GAG TCC TTT TTG ACT TTC | ACG TTG GAT GCT GCG AGA GGC TTT TGT CAG | CCT TTC CTG CAA CGC |
| W2 | URB_2730(ex7) | ACG TTG GAT GAT TCA ATG CAA CTC CGG AGG | ACG TTG GAT GGC CTG CAT ACT CAT CTT CTG | TTC TGG GCA GCG CAT |
| W2 | PAPSS2_3'UTRa | ACG TTG GAT GAG CAG GAG GTC CTA GAT TTC | ACG TTG GAT GAG CTG GCA AGA CTT AAG CTG | TGG CAG GTA AAC TGG A |
| W2 | UGT2B17_197(ex1) | ACG TTG GAT GCT TGA CTT ACA GAT GCA GGG | ACG TTG GAT GAT CAT CAG CTG CCA TTC TTG | GCC ATT CTT GTT GAT CC |
| W2 | Bap1_3'UTRb | ACG TTG GAT GAC GGG ATG AGC AGC CTG TTG | ACG TTG GAT GGA ACC AGG AAG TTT ACA GGC | GAT CTC TGC TCA GGT CA |
| W2 | AKR1C3_in2b | ACG TTG GAT GTG CCC TGC AGT TAC TAA TAG | ACG TTG GAT GTG CAC TCT GCT TCT GTG GAA | CTT CTG TGG AAG AGT CAA |
| W2 | MMP1_in5a | ACG TTG GAT GAC GCT TCT TAA AGA GTA CAG | ACG TTG GAT GTC CAG GCC ATC TAT GGT GAG | AAT GGT GTC ACT GGA ACA |
| W2 | UGT1A1_in3b | ACG TTG GAT GGA GGA GGG TTT GAT GTA TAG | ACG TTG GAT GAC GAT CCT TTA GAA CAG CCC | GCC TAA GCC ACT TTC TTA A |
| W2 | UGT1A1_in2a | ACG TTG GAT GTC TGT TGG TTA GGA CAA TGG | ACG TTG GAT GTT CTG CAC ATC AAA GGA AAG | CTG AGT GGG TAT AGA GCT A |
| W2 | FDX1_3'UTR | ACG TTG GAT GAC CTG GGC CCT AGC CCC AA | ACG TTG GAT GAA GGG GAG GCT CGG CGA GT | ATG GCG GTG CGC GGG TCT G |
| W2 | CTNND1_3'UTRa | ACG TTG GAT GCC AGT TGC ATT GAA CAG AGC | ACG TTG GAT GCA GCT GCT ATG CGG TTG ATT | AGC CCT TTC CCT TAT TGG TC |
| W2 | NGFIB_1195(ex4) | ACG TTG GAT GAG GTC ATA GAA CTG CTG CAC | ACG TTG GAT GCT GCC TTC TAG TTC CAG GAG | CCC ACC ACT TCG GGA AGG AG |
| W2 | MMP1_in3a | ACG TTG GAT GAT GTA GCT GCA AAG CCG AAG | ACG TTG GAT GTA AGC TCT TCT TGT GGC GTG | TCT CAC CTT AAC TTC ACC TTG |
| W2 | RALBP1_3'UTR | ACG TTG GAT GAA GCC CAG CCG TTT CTA TTG | ACG TTG GAT GGA TTT CAG TTG TCC CTG TGG | ATC TGC GGA ACT GCC GCT GGA |
| W2 | HSD17B4_in18b | ACG TTG GAT GTG GAA AGG TAG GAA GTA CTG | ACG TTG GAT GCA GTC CTG TCC TAG AAC TTG | CCT AGA ACT TGA ACT AGG AGG |
| W2 | SULT1B1_746(ex6) | ACG TTG GAT GGA TGG TTA GAT TTC TAG AG | ACG TTG GAT GGG GTT GTC CTT CAT CAT TTC | CGA TCC AAG ATT TCA TCA TTC A |
| W2 | RNF14_3’UTR | ACG TTG GAT GCA CCT GAA CAA GCT GAA CTC | ACG TTG GAT GAC AGA ATT CAG AAG GTC AGC | AGT AAG AAG GTC AGC ACT GGA G |
| W2 | CYP2C49_1095(ex7) | ACG TTG GAT GCC GTG TTG CAT GAG ATA CAG | ACG TTG GAT GTC TCT GAA TTT CGT GTC CCG | GAA AGG ACT GTG GCA TGG GGT A |
| W2 | SRD5A2_3'UTRd | ACG TTG GAT GCA CTG CCC TCA ACT CAA AAC | ACG TTG GAT GTT CTC TGA ATG GCC CAA GTC | TTC AGT ATA AGA CAG CTC TGT AT |
| W2 | DHRS6_3'UTR | ACG TTG GAT GAG GAT GGG TGA CTG TTC TTC | ACG TTG GAT GGA TTT CAG GAT CTC CTT GCC | GAT GTG CCT GGG AAG GCA GTC ACT |
| W2 | SOX9_in2a | ACG TTG GAT GAA ACT AAA GCC TTG GCA CAG | ACG TTG GAT GTT GGT GGA CCC TGG GAT TG | ATG TGA TTG CCT GCA GGC AAA GAG |
| W2 | CYP2C49_754(ex5) | ACG TTG GAT GGA GGG TTG TTA AGA TTC GGG | ACG TTG GAT GGG AGA GCA CAG AGC ATT TTC | AGG GTG GAA AAA GTA AAG GAG CAT |
| W2 | Hsp70_1476(ex1) | ACG TTG GAT GTG GTG ATC TTG TTG GCC TTG | ACG TTG GAT GAG GTG ACC TTC GAC ATC GAC | CCC ACC CTG AAC GTC ACG GCG ACG G |
| W2 | CYB5_3'UTR | ACG TTG GAT GGG GTG ATA TAC GTC ATT GAT | ACG TTG GAT GGC CAT GGA GTT CTA GAG AAA | GAA TTT TTC TAA ATA ACA GAG CAT C |
| W2 | UGT2B7_in4a | ACG TTG GAT GTA TTT CCA GCC TCT GAG GAC | ACG TTG GAT GGC CAT GAG AAA TGT GAC CTG | TCC CGG CTT TAA CAA AAT GCC ATC TCT |
| W2 | RAD23B_3’UTR | ACG TTG GAT GAC TGG GTT TCT GAT ATT TG | ACG TTG GAT GCC ACC TTC TCT TTT TCA AAG C | CCA CGC AAA TAA TCC TAA AGA CTT TAA |
| W2 | Hsp70_1748(ex1) | ACG TTG GAT GTG CTC AAA CTC GTC CTT CTC | ACG TTG GAT GAA GAA GGT GCT GGA CAA GTG | GTT CCG GAG GTG ATT TCC TGG CTG GAC |
| W2 | MMP1_279(ex2) | ACG TTG GAT GGA AAC CAG ACG CTG AAA CTC | ACG TTG GAT GGA GTG AGA ACA AAC TCA GCC | GGA TAA CAA ACT CAG CCA CGT CAG GAA C |
| W2 | HSPCA_3'UTR | ACG TTG GAT GGC ATG ACA GTT CCT TAA GGG | ACG TTG GAT GAA GGA CAA GCT CGG CTT TAG | TTT GAA AGC TCG GCT TTA GTG CTC AAA T |
| W2 | DHRS8_3'UTR | ACG TTG GAT GAA AGC CTT CAG ATG AGC CAC | ACG TTG GAT GTC TTG GGA ACT GAT CTT AT | GTG GCT TGG GAA CTG ATC TTA TGG AAA A |
| W3 | CYP21_in8b | ACG TTG GAT GTT GCC ACA CCG AGC CAC TC | ACG TTG GAT GAG TTA GAG GCC ACC AGG AC | CTC ACC CCT GCT CCT |
| W3 | CYP2D6_1287(ex7) | ACG TTG GAT GGC TTC TAT CCA GAG CAC TTC | ACG TTG GAT GCT GCT GAG AAG GGC ATG AAG | TCC TGC TTG GTG AAG |
| W3 | HYAL1_748(ex1) | ACG TTG GAT GAT GCT GCA CAT ACA GCT GAG | ACG TTG GAT GCC CTC TAT CCT AGC ATC TAC | CAG CAT CTA CCT GCC C |
| W3 | HYAL1_83(ex1) | ACG TTG GAT GTC CTG AAC TTG CTC AGC GTG | ACG TTG GAT GTT GCA TTC CAG ATG GTG GTG | GGT GAA TGG CCG GTT G |
| W3 | SARG_3'UTR | ACG TTG GAT GGC ATT GTG TAA AGC CTC TGG | ACG TTG GAT GCC AAT CTC AGT TTG CAT GTG | TTA TGT GAC TGA CCC CC |
| W3 | MIS_in1 | ACG TTG GAT GTC TCT TGG TCT GAA GCT CTC | ACG TTG GAT GGA AGG TCC AGC AAC AGA CAA | GCA ACA GAC AAA TAC ACA |
| W3 | CYP11B1_in1b | ACG TTG GAT GGG TGT CGT GTG GGG GAG GG | ACG TTG GAT GTG TGT GTG TCT GCC CTC CTG | CAT GCA GGG AGC CTC GCC |
| W3 | CTNND1_3'UTRb | ACG TTG GAT GGC TCT GTT CAA TGC AAC TGG | ACG TTG GAT GTT TTC TCC TAC CCC ATA GCC | CCC CCA TAG CCT CAA TAT A |
| W3 | NGFIB_in4 | ACG TTG GAT GTC AGG GGA GCT CAA GGG CA | ACG TTG GAT GTC ATT CTC CGT TTG GCC TAC | TCC AGG TGA GCC CCC CTC C |
| W3 | AKR1C3_in4c | ACG TTG GAT GTT CTC TAG AAA CGT GCC AGC | ACG TTG GAT GTT CGG CAA GTC GTG AAC TTC | CTT CAG AAG GAC TGT TAG G |
| W3 | Hsp70_-127(prom) | ACG TTG GAT GAT GTC GCT GGC CTG CTC TCT | ACG TTG GAT GTT CAG AGC TCT CGT CTG GGA | GGT AGG GAT CCC GGT TTC G |
| W3 | CYP2C49_1083(ex7) | ACG TTG GAT GTC TCT GAA TTT CGT GTC CCG | ACG TTG GAT GCC GTG TTG CAT GAG ATA CAG | TAC AGA GAT ACA TCG CCT TA |
| W3 | Hsp70_1537(ex1) | ACG TTG GAT GTC ACC ATC ACC AAC GAC AAG | ACG TTG GAT GTC CTC CGC TTT GTA CTT CTC | CCC AAT GCG CTC GAT CTC CTC |
| W3 | HYAL2_583(ex1) | ACG TTG GAT GGA ATT TGA GTT TGC TGC CCG | ACG TTG GAT GAC AGA TGC TGA GGC CGA AAC | GAA TAA ACG CCT TGA CAA ATC |
| W3 | EGFR_in2b | ACG TTG GAT GGC AGA GTC TCT CTT CTG AGG | ACG TTG GAT GTG TCA ACC ACT CTT GTG CAG | CCT GGT CGC AGG CTC ATC TTA C |
| W3 | AKR1C3_in4a | ACG TTG GAT GAC ACA GTG GAT CTC TGT CGC | ACG TTG GAT GAA GTG AAG GAT TTC CCC CTC | GCT ACC CCC GGA CTC TCT CCT CC |
| W3 | TSPYL4_3'UTR | ACG TTG GAT GCC TCC TTG CTG CAG TGT AAG | ACG TTG GAT GTT CAG TTT CCA CAG CCA AGG | GTT GCC ACA GCC AAG GAC CCT CC |
| W3 | UGT1A1_in2b | ACG TTG GAT GCT TAA TGA GTG GGT ATA GAG C | ACG TTG GAT GTC TGT TGG TTA GGA CAA TGG | TTA GGA CAA TGG ATT CAT TTC TT |
| W3 | CYP2C19_3'UTR | ACG TTG GAT GGG AGA GCA CAG AGC ATT TTC | ACG TTG GAT GGA GGG TTG TTA AGA TTC GGG | CCT TTT TCC AGA ATA AAA GAC CTA |
| W3 | EGFR_in2a | ACG TTG GAT GTC TGT CTT GCA GGT TTT TCC | ACG TTG GAT GAA CAC TGG ATC CCT AAC CTG | GTG ATG AGC CGC CAG GGA GCT CCA |
| W3 | HYAL3_571(ex1) | ACG TTG GAT GCA CAA GCT GGG TAG TGA TAG | ACG TTG GAT GAC TGA TGG AGG ACA CAC TGC | GGA GAA CTG CGG CTA GGC CGC ATG |
| W3 | UGT2B17_420(ex1) | ACG TTG GAT GGT AAA GAT GCT GTC TTG AAC | ACG TTG GAT GAT GGG ATC TGC AAG GAT GAC | ATG ATG ACA TCA AAC CTT GAT TCT T |
| W3 | CYP2E1_1424(ex9) | ACG TTG GAT GGA CAC AGA GTT TGT AAT GGG | ACG TTG GAT GTG TTG ACC CCA AGG ATA TCG | CCC CAG GAT ATC GAC CTC AGC CCC AT |
| W3 | CYP21_in6a | ACG TTG GAT GAA AGA AGC CAC CTA GCT CTG | ACG TTG GAT GTC GTG GAG AAG CAG CTG AG | AGA TGC GGC ACA AGG TGG GAG CCA TG |
| W3 | UGT2B7_in4b | ACG TTG GAT GGT CCT CAG AGG CTG GAA ATA | ACG TTG GAT GTA TCT ATC ATT CTC CTA GC | CTT ATC CTA GCA TTT ATT ATT GAA ACT |
| W3 | NCOA4_3’UTR | ACG TTG GAT GAG TTC TAA ATG GTA TCT GGG | ACG TTG GAT GTG CAG TCC CAG TGT CAT TAC | GGG TAT TGA ACC ATA GCT GAA ACA AAC |
| W3 | HYAL2_in1a | ACG TTG GAT GGG GAA CTC TGG GCA AAT TTC | ACG TTG GAT GTC TGT ACG CCT ATG AAA GGG | CCC CTT GGC ATC ATT ATC TCT ATT TTG C |
| W4 | EGFR_in12 | ACG TTG GAT GAA TGG TGC AGG CCC AGC CTC | ACG TTG GAT GTG ACT GTA GCT AGC CAC ACA | ACA CGC ACT CAC AGG |
| W4 | CYP21_in9 | ACG TTG GAT GAG TTC CGG CCA GGT GTG TG | ACG TTG GAT GGA CCA AAG GAC AGA CCC AG | CAG ACC CAG GTT CAG |
| W4 | Hsp70_859(ex1) | ACG TTG GAT GAA GAG GAC CCT GTC GTC CA | ACG TTG GAT GTG GAC GTG TAG AAG TCG ATG | TCC GAT GCC CTC GAA CA |
| W4 | CYP3A4_3'UTR | ACG TTG GAT GTT CTT CTG AAG GTT GTG CCC | ACG TTG GAT GTG CAG CTT CCT TGA GGA ACC | CTT GAG GAA CCA AGC CC |
| W4 | Bap1_3'UTRa | ACG TTG GAT GTA TGT ATT CTG CTG CCC AGG | ACG TTG GAT GTC CAC AGC TGG CTG TGG AG | GGC TGT GGA GCG GTA GG |
| W4 | CYP21_in6b | ACG TTG GAT GCG GCC ACC ATG CTC TCC TG | ACG TTG GAT GAG TCC TGG ACC TGT TGC TC | CGG ACC CCG AAC CCC CAG G |
| W4 | ALB_1103(ex9) | ACG TTG GAT GTC TTT GGC ACA GCA GTC CTC | ACG TTG GAT GAC TCT GTC TCA TTG CTG CTG | GTG CTG AGA ATT GCC AAG A |
| W4 | CYP21_in8c | ACG TTG GAT GAC TCG GCC TAG CAG GTG ACT | ACG TTG GAT GGA AGG GTG GAG TTA GAG GC | CCC CCC CAG GAC CCC AGT GG |
| W4 | EGFR_3'UTRa | ACG TTG GAT GAA GGT CCG AGC GTT AAC GTG | ACG TTG GAT GAT GGC ATT GGC CCA AAA CTG | CGA CGG CAG CTA CAT AGT CC |
| W4 | CYP2C49_1251(ex8) | ACG TTG GAT GAA GAA TTC CCC AAC CCA GAG | ACG TTG GAT GAA GGC ATG AAG CAG TCA CTC | GCA GTC ACT CTT CTT AAA GTT |
| W4 | Hsp70_1258(ex1) | ACG TTG GAT GTG ATG ACG GCG CTG ATC AAG | ACG TTG GAT GGA GTA CGT GGT GAA GAT CTG | ACT TCG TCT GCT TGG TGG GGA |
| W4 | UGT1A10_3'UTR | ACG TTG GAT GTC TGG ACC ACA CTT GTC ATC | ACG TTG GAT GGG ATC CCA TTT CTA AGT AGG | GCA GAT TGA AAG AGG GTC CTA |
| W4 | HSPCA_2175(ex9) | ACG TTG GAT GTG GTT GTG TCG AAC CGG TTG | ACG TTG GAT GTC TCT CCA TGT TTG CTG TCC | CCA CTG TTT GCT GTC CAG CCG TA |
| W4 | IQGAP2_3’UTR | ACG TTG GAT GAT TTA GAC AGT TCT CAG CCC | ACG TTG GAT GGA ATT GCA CAA AAG TTC TC | GCA CAA AAG TTC TCT TTT AAA AT |
| W4 | MMP1_in5b | ACG TTG GAT GGC TTC TTA AAG AGT ACA GCC | ACG TTG GAT GTC CAG GCC ATC TAT GGT GAG | GGT GAG TAT AAA GAA AAA CAC TA |
| W4 | CRSP9_504(ex1) | ACG TTG GAT GTG GAA CGA GTC ATT GAG ATG | ACG TTG GAT GTC AGC TTT TAC TCT CAT CCC | GGG ACA GTC ATC AGG CAA AGA AGC |
| W4 | MMP13_in3a | ACG TTG GAT GGG TAC CTA GTG CAG GTA CTT | ACG TTG GAT GCT GCT CCT TAA TTG GTT TGA | TTA GTC ATG CAT TAC TTT TTA TAG A |
| W4 | HSD11B1_793(ex6) | ACG TTG GAT GTA ATA CAC TTC CTC TTG GCG | ACG TTG GAT GCG TAC AAC CAT CTC CAA AGG | CTC AAA CCA TCT CCA AAG GAA GAA TG |
| W4 | MMP1_in1 | ACG TTG GAT GCA ACC ATG CAA TCT AAA CAC A | ACG TTG GAT GGC TGG ATG GGA AAT CTT TCT | AAG ATT GAC AGT ATA AAG TTA TAT TT |
| W4 | AKR1C3_in2a | ACG TTG GAT GCC AAC AGA ACT AGG ATC AGC | ACG TTG GAT GTG AGG GAG GTG ATA AAT GAG | GAG GTG AAG AAA TTT TAG AAA GTT AC |
| W4 | SRD5A2_3'UTRe | ACG TTG GAT GGT CTT AGT GCC TCT GTC TTC | ACG TTG GAT GAA CCC ACC CCA GTT TTC TAC | GCC AAG TTT TCT ACG TTA GAC TAT ATC |
| W4 | Hsp70_-46(prom) | ACG TTG GAT GAA ACC GAA CGC GCC GCT TCT | ACG TTG GAT GAG CTG CTA AGA CTT CCT CTG | GTC GGT GAG CGC AGC CTT GGC AGA TCC G |
| W5 | NGFIB_1374(ex5) | ACG TTG GAT GTG CCC TTG AGC TCC CCT GA | ACG TTG GAT GAG GCC TGA GCA GAA GAT GAG | GAT GAG CTT CCC CTC |
| W5 | CYP2D6_1276(ex7) | ACG TTG GAT GTG CTG AGA AGG GCA TGA AGG | ACG TTG GAT GCC TTC CGC TTC TAT CCA GAG | AGA GCA CTT CCT GGA |
| W5 | NGFIB_in3 | ACG TTG GAT GAC CAC GTA CAG CCA TGT CAC | ACG TTG GAT GAA GCA AGT GTG GGT GGA ATC | CTG CTC TGC TCT CAC C |
| W5 | EGFR_3'UTRc | ACG TTG GAT GAA GCC ACT TTG AAC CCT TCC | ACG TTG GAT GAG TAC CGT GGA GAT ATT TGG | GAG GAA TTC TAC CCT CC |
| W5 | UGT1A1_in3a | ACG TTG GAT GAG GGC TGT TCT AAA GGA TCG | ACG TTG GAT GAT GCC CCA CTG GGT AAA AAG | TGG TAA AAA GCC GTG TG |
| W5 | PPP1R1A_291(ex5) | ACG TTG GAT GTG AAC ATC ACC TGG GGC AAC | ACG TTG GAT GTG TGG TTG TGA TCC CAG GTG | TCC TTC GGG CTC CTC TCC |
| W5 | EGFR_3'UTRb | ACG TTG GAT GAC TTC CAG CTG AGA AGT GCC | ACG TTG GAT GAG ACA AAG GAA TGT CCC TGC | GAT GCA ACT TCC AAG GAC |
| W5 | FTH1_5'UTR | ACG TTG GAT GTC ATG GCG GGA GGC TGA GGA | ACG TTG GAT GAC CTC TTC ACA GCG CCC TC | GAC CGC TCC CGC GCC GCT G |
| W5 | AKR1C3_in4b | ACG TTG GAT GGT CCT TCT GAA GTT CAC GAC | ACG TTG GAT GGG GAA ATC CTT CAC TTC TTC | GAC AAT GGG CTA TGG ACT G |
| W5 | HBS1L_1994(ex17) | ACG TTG GAT GAG CAG CTA TGG TAG AAC CAC | ACG TTG GAT GCA GTG GCT CTT GAG CTA TAC | AAG ACT TTA AAG AGC TGG G |
| W5 | PAPSS2_3'UTRb | ACG TTG GAT GGT TAA TAG GTG CAG CCT CTC | ACG TTG GAT GCC TGG CAA TGT TGT GGA AAG | ATG TTT CTC CTC TTT GCT GT |
| W5 | HBLD2_3'UTRa | ACG TTG GAT GTT CTC CAA TAA GCC CCA GAG | ACG TTG GAT GAA CAT CTG ACT CCT CAG GAC | CCT AGG ACT GCT CGG GCC GT |
| W5 | FTH1_3'UTR | ACG TTG GAT GAG AAA AGG TGA CGG TAA CCC | ACG TTG GAT GAG TGA GAG CTA AGC CCT AAG | CCA TCC CTA AGC TGG CCT CCC |
| W5 | CYP11B1_in1a | ACG TTG GAT GTT CCT GCC CCT CCC CCA CA | ACG TTG GAT GCT CAC TGT CCG TCC CTA CTA | GGT TCT GGG TCC CTC CCT GCT |
| W5 | MMP13_in2 | ACG TTG GAT GGC ACA AGC TAC AGA TGA CAG | ACG TTG GAT GAC AGG ACC TAC TTA ATT TC | GGA CCT ACT TAA TTT CTA GTT TA |
| W5 | SRD5A2_3'UTRc | ACG TTG GAT GAC GGG ACC CCT TAG TGT TTC | ACG TTG GAT GCC TCT GCA TAA AAG CAG ACC | AGT TTT AAT AGG AAA TCA GGA TAG |
| W6 | CYP2E1_in1b | ACG TTG GAT GTT AAC GCT CTG CCA GCT GTG | ACG TTG GAT GTT AAA GGG CAT GTT TCC TCC | TCC TCC CCA AGT CCT |
| W6 | SOX9_in2b | ACG TTG GAT GAG TTT TAG GAA CCC GTC CAG | ACG TTG GAT GTC ATT GGG CGG TTT ATC TCG | TTA TCT CGG GTG CAG C |
| W6 | CYP2E1_in6 | ACG TTG GAT GAG CTT GAC TGA TCT GGA TGG | ACG TTG GAT GGC ACA ACT TCA AGA AGG TCC | AGG TCC AGT TCT TCT GAG |
| W6 | HPGD_3’UTR | ACG TTG GAT GGT GCC AAT TTC AAA AGT GCC | ACG TTG GAT GAC TGT TTT CTT CTC CTC AAC | CTT CTC CTC AAC AGC TAT A |
| W6 | HSD17B1_3'UTRa | ACG TTG GAT GAG TGG TCC TCG ATC CCC AAG | ACG TTG GAT GAA GTC ACA AGC CAA CTC TCC | CCT CTC AAG TCC GAC TAG C |
| 1 | HNF4A_in4c | ACG TTG GAT GCG TTG ATT CCA GAG ACA GGG | ACG TTG GAT GAC CGA CTA CAT AGA GAG GGC | CCC AGC ATT TTC CTC C |
| 1 | CYP2A19_108(ex1) | ACG TTG GAT GAT GGT CTT GAT GTC CGT CTG | ACG TTG GAT GTA GTT CCC GAT GAA GGG CAG | AGG GCA GCG GGG TGG G |
| 1 | CYP2B6_in7a | ACG TTG GAT GCC CTG AAG AAA GGT CAC GAG | ACG TTG GAT GGG CTT TAT CAC TCA GTC TTG | CGT TTC ATT CTG GGG GC |
| 1 | CYP2B6_308(ex2) | ACG TTG GAT GTC TGA GGG CCT TAC CAT ATC | ACG TTG GAT GTC TCT GGC CGA GGG AAA ATC | GAG GGA AAA TCG CTG TG |
| 1 | HNF4A_in4b | ACG TTG GAT GAG TTC GGA GAG GAG GGC TGT | ACG TTG GAT GGT TCT AAG GAG TTG GTG GAG | AGT GGA AGG TGG CCA GGT A |
| 1 | CYP2B6_3'UTRi | ACG TTG GAT GAC GTT CTA CAC ATA CAC CCC | ACG TTG GAT GTC CTT CCG CAG CTC TTC TGA | GGA AGC TCT TCT GAA GCC TC |
| 1 | AKR1D1_in6d | ACG TTG GAT GGG TGT GTG ATT TTA AGG AGG | ACG TTG GAT GTT CAC AGG CAG AGA ACA ATC | TGG TTT TAT TAG CTT GTG TTT G |
| 1 | STS_1513(ex10) | ACG TTG GAT GAG TTA ACG GGT CTG TCT CTC | ACG TTG GAT GTG TCA CGG GCA TTC CAT CAC | CAC CAC GAC CCT CCT TTG CTG TTT GAC |
| 1 | HSD3B_-15(prom) | ACG TTG GAT GTC CCC AGT GTT TTC TGG TTC | ACG TTG GAT GCC ATC CAG CCA TTG CTA AAC | ACC ATC CAG CCA TTG CTA AAC CTG GGC |
| 1 | SRD5A1_646(ex4) | ACG TTG GAT GTC GTG GAG TGG TGC GGC TA | ACG TTG GAT GAG GAC GCA GAA GGT GAA GAG | AAC GCC GAG CCC TGG A |
| 1 | PXR_in5c | ACG TTG GAT GAG TCC CTT GCA GGA TGA TGG | ACG TTG GAT GTC CGG TGC CAA GCA GTT TAC | GAA AAT AGC CCC ATG CC |
| 1 | CYP1A2_310(ex1) | ACG TTG GAT GTG CTC AGC GGC TGG ACA CAT | ACG TTG GAT GAT CAG TGA CCA GAG TGA AGC | GGT GGC CGT CCC TTG AAA TC |
| 1 | AOX_in13 | ACG TTG GAT GAG CAA AGG CAT AGG TCA CAG | ACG TTG GAT GAT CTG AGA TGC AGC TAC CAG | CCT ACA CCA CAG CAC AGC AAT |
| 1 | AKR1D1_672(ex6) | ACG TTG GAT GCT TCT TGA GGA GAC ACA CAG | ACG TTG GAT GGT CAT TAT TGC CTA CAG CCC | GGT CTA CAG CCC TTT GGG AAC |
| 1 | STS_1307(ex9) | ACG TTG GAT GTT GAC GGA CGT GAC CTG ATG | ACG TTG GAT GCG GCG TTC AGA TAG AAG TTG | GGA AGA GAA ACT CAT GAT CAG AA |
| 1 | GUSB_in2c | ACG TTG GAT GAG ATG AGG CAA CCC TCC CAA | ACG TTG GAT GAG CTG TCA CGT GAC AGC CT | GTG ACA GCC TGC TTC CT |
| 1 | STAR_in4 | ACG TTG GAT GAA CCA CCC CAG CTT GCA AC | ACG TTG GAT GCG CGG TAC GGT TAG AGA CA | GGT TAG AGA CAG GTG GG |
| 1 | CYP2B6_in7b | ACG TTG GAT GCA GTC TTG TTT CAT TCT GGG | ACG TTG GAT GCT TCT GTG CCC TGA AGA AAG | GGG AAA GGT CAC GAG ATT AGA |
| 1 | CYP1B1_216(ex1) | ACG TTG GAT GTT GGA AAC GCG GCG TCT ATG | ACG TTG GAT GTC TGG AAG ACA TCG CCG TAG | CCC GAG CCA GGC GAG CGA AAG A |
| 1 | CYP51_1035(ex7) | ACG TTG GAT GGG CCA GAG ACA AAA CAC TTC | ACG TTG GAT GGG TCA TAA GTT AAT GGT GGC | TTT AAT GGT GGC AGA TCC TCT CC |
| 1 | PXR_in4b | ACG TTG GAT GAA CCC TAG GAG AAA ACA GCC | ACG TTG GAT GAG GGA AGG TCC CAA ATG ATG | CTG GCC CTG CCC TCC ACT CAC CGC A |
| 1 | HNF1_in3c | ACG TTG GAT GAC AGA AGA ACC CTC TCC TTG | ACG TTG GAT GAG TCT GCC AAC CTC AAA CAC | AAC CTC AAA CAC TCA GGT AGA TCT CTC |
| 1 | PXR_in5a | ACG TTG GAT GAT GTA AAC TGC TTG GCA CCG | ACG TTG GAT GTG CTC TGT GAC CTC CAC TG | CTG AGG CCC TCT CTG C |
| 1 | CYP11A_150(ex1) | ACG TTG GAT GTG CCC TAC CTC TTT TCT CAG | ACG TTG GAT GTG GTC TCT GGA ATG CTG ATG | AGC CCA GGG TTA GGA G |
| 1 | CYP1A2_581(ex1) | ACG TTG GAT GGC ACT TTG ATC CCT ATG ACC | ACG TTG GAT GAA CAT CTC CTC ACT GCT CTG | GAC ACG GCC CAC TGA CA |
| 1 | CYP2B6_3'UTRb | ACG TTG GAT GAG CCT CAG TTT CTT CCC TTC | ACG TTG GAT GTA AGG ACC CGG CTT GTT CAC | AAG GAT GTG GCA CAA GTC |
| 1 | CYP1A2_212(ex1) | ACG TTG GAT GTG CTG ACC TTG GGC AAG AG | ACG TTG GAT GAG CAC AGG GGT GCA GCC AAT | TTC AGC ACA TCT CCA TAG C |
| 1 | HNF1_in3a | ACG TTG GAT GTT GTA GTG AGG ATG AGG GAG | ACG TTG GAT GAG TGT CTG AAA CCC AGC TCC | GGC CAT TTC TGG TGC CCC CC |
| 1 | PXR_in4d | ACG TTG GAT GGT AAT GGC AAA CCT CTC CTG | ACG TTG GAT GCA TGG CAC TGT ATC ACC TTC | CCC TGT ATC ACC TTC CCC TTC |
| 1 | PXR_in1 | ACG TTG GAT GTT CTC AAA GAC TGG CCT CTG | ACG TTG GAT GAG GGT GGT CTG AGA ATT TGC | TTT GCA TTT CTA ACA GGT TCA |
| 1 | HSD3B_825(ex3) | ACG TTG GAT GGG AAG GCT CCT TCT GGA ATC | ACG TTG GAT GCT CCC CAA AGC TAC GAT GAC | AAA TTA CAC GTT GGG CAA GGA |
| 1 | CYP2B6_3'UTRf | ACG TTG GAT GTC CCC ACC TCT GTG GAG AAG | ACG TTG GAT GAT GTG AGC AAG GGA AAG AGG | CCA GGC GGG AGA GAG GCA GCG |
| 1 | AKR1D1_in6b | ACG TTG GAT GTT CAC AGG CAG AGA ACA ATC | ACG TTG GAT GGG GTG TGT GAT TTT AAG GAG | TGT GTG ATT TTA AGG AGG TAA CA |
| 1 | TR4_in12b | ACG TTG GAT GAG GCT GTA GTC CTG AGT AAC | ACG TTG GAT GGA GGG CCA AAT GTA TAC TCA | GGA GTT AGG ATG AGA TCT ATT CAG |
| 1 | HSD17B8_in5 | ACG TTG GAT GAA AGA GGA CAG AAC ACG GAC | ACG TTG GAT GAG CTC GGA CGG TTG GTC AG | GGT GGC ACG GGA GGT TTT GCT GAG GC |
| 1 | PSDR1_in3a | ACG TTG GAT GAG GAG GAA GTG ACC TAT GAG | ACG TTG GAT GTA TAG CTG AGC CTT GCT CAC | TTG CTC ACC ACT GCT CC |
| 1 | CYP2B6_3'UTRg | ACG TTG GAT GTC GTA GGA ATG TCC TGT CTG | ACG TTG GAT GGG GAA AGG AAT AGA CCT CTG | CCG AAG AGC TGC GGA AGG ACG G |
| 1 | RLN_206(ex1) | ACG TTG GAT GTC TTT GGT GAT GGA GGA TGG | ACG TTG GAT GAA CTG CTC TCA GCC TGG AAG | TTC AGC TGG AAA CTG GAC CCC CG |
| 1 | HSD3B_271(ex2) | ACG TTG GAT GTC ATC CAC ACT GCC TCT ATC | ACG TTG GAT GTT GAC CTT CAT GAC GGT CTC | CCC ATG ACG GTC TCT CGC CCA ACG G |
| 1 | TR4_in12c | ACG TTG GAT GGA AAG CTT CAC CTG CAG ACC | ACG TTG GAT GTA GAG CAG CCT TCA GAG TTC | AGA AAT TTG TCA GAA GTT TGA AAA TA |
| 1 | CYP51_819(ex6) | ACG TTG GAT GAA GGT TTG GAG AAT GTC ATC | ACG TTG GAT GGA GCA CAT CGA GAG ATC AAG | ATC TAA GGC AAT CCA GAA ACG CAG ACA |
| 1 | HNF1_in3b | ACG TTG GAT GAG TGT CTG AAA CCC AGC TCC | ACG TTG GAT GTT GTA GTG AGG ATG AGG GAG | TGT TTG GGT CAG TGG G |
| 1 | INSL3_331(ex2) | ACG TTG GAT GTC AGG CCT CTG GCC ATC AC | ACG TTG GAT GTC AGC AAG TCT TGC CGG GTG | GTG GCG GGC TGG GTT GG |
| 1 | HNF4A_in4d | ACG TTG GAT GAG TGG CAA AGA TTC GAA CCC | ACG TTG GAT GAA ATG CTT GGT TTC CAA GGG | TGG CGG GCC CCG CAG GAC |
| 1 | CYP2B6_3'UTRh | ACG TTG GAT GGT TCA CGG ATG TGG CAC AAG | ACG TTG GAT GCC CCT TTT AGC CTC AGT TTC | GGG CCT CAG TTT CTT CCC T |
| 1 | CYP2B6_1078(ex7) | ACG TTG GAT GGT GTC TTT AGT GAC CGT GTG | ACG TTG GAT GTG GAT GCG GTC ATT CAT GAG | GAA GAT GCA GAG ATT TGG G |
| 1 | PXR_in5b | ACG TTG GAT GTG CTC TGT GAC CTC CAC TG | ACG TTG GAT GAT GTA AAC TGC TTG GCA CCG | CAC TTC TCA ATA AAC AGG AG |
| 1 | CYP2B6_in2a | ACG TTG GAT GAT TGG AGA GGG TGT GTC TGG | ACG TTG GAT GGA GGC TTG GAA TGC AGA GGA | GCT GGG AAT GCA GAG GAA GTT GG |
| 1 | CYP2B6_3'UTRe | ACG TTG GAT GCA TCT GCA AGA TGG GCA TGA | ACG TTG GAT GTC CTT GGG CTC CAT GAT CTG | GGG GAT CCA TGA TCT GGG CAT ACA |
| 1 | AKR1D1_in6a | ACG TTG GAT GAA TCA CAC ACC CAA GCA GAG | ACG TTG GAT GCT GTG TGT CTC CTC AAG AAG | GGG GAG CTA GTG CAA ATT GCT TGA T |
| 1 | CYP1A1_1527(ex6) | ACG TTG GAT GTG ACC ATG AAG CAT GCC CAC | ACG TTG GAT GAG AAA GTC TAG GTT GCA GGG | AAC CGA AGA GCG CAC ATG CAT CTG GAC |
| 1 | HNF4A_in4a | ACG TTG GAT GCA GGG ATG GAG CTG AGA TTC | ACG TTG GAT GAG GTC CTG TCC CAG CAG GTA | ACC CAC CTG GGG ATC TCT |
| 1 | STS_1554(ex10) | ACG TTG GAT GTG CAT CAC CTC CAG GAT CTC | ACG TTG GAT GAC CCT CCT TTG CTG TTT GAC | TCG ACC CGT TAA CTC CGA C |
| 1 | TR4_in12a | ACG TTG GAT GAA TGG AAA GGA CAT GCT CGG | ACG TTG GAT GAC TGG ATG GCA GTA CAG TTG | GCA GTA CAG TTG TAT CCC CTC |
| 1 | AKR1D1_in6c | ACG TTG GAT GTT CAC AGG CAG AGA ACA ATC | ACG TTG GAT GGG TGT GTG ATT TTA AGG AGG | GCT CTT CAA CTC ATT CCC CCA T |
| 1 | CYP3A29_1201(ex9) | ACG TTG GAT GTT CAG GAC GGA ACT CCT CAG | ACG TTG GAT GTG GCG TGT TCG TTC CCA AAG | GGT TTC GTT CCC AAA GGG ACC GTG |
| 1 | CYP2A19_1376(ex9) | ACG TTG GAT GGA GAA GGT CTG GCT AGA ATG | ACG TTG GAT GAG CTG CGG AGA CTT GAG GTG | TCT GCG GAG ACT TGA GGT GGA AGT T |
| 1 | CYP2B6_3'UTRa | ACG TTG GAT GAT TGG AGA GGG TGT GTC TGG | ACG TTG GAT GTG GAA TGC AGA GGA AGT TGG | GGG GCA GAC TCT GGG TCC CCC TAA AC |
| 1 | PXR_in3 | ACG TTG GAT GTG TCC TAC AGC TTG GAA GAC | ACG TTG GAT GAC GTG CAC ATC CTG ACC TG | ATC CTG ACC TGG CTC CT |
| 1 | CAR_in1 | ACG TTG GAT GCT GTG AGG ACA GAG ATG TTG | ACG TTG GAT GAG ACC ATG TCA TTT CAG CCC | GCC ACC TTA CTC GCT GCC |
| 1 | CYP1B1_93(ex1) | ACG TTG GAT GTA CGA TCA CCT GCT GCC CAT | ACG TTG GAT GTC AAC AGC CAC TGG CCC AC | AAC TGG CCC ACG TGC ACC GC |
| 1 | PXR_in4a | ACG TTG GAT GTG AAG CTG ATG GGT TGC AAG | ACG TTG GAT GTT TCT CTC CAG GTG AGA GTC | GGG CCA ACC GTG GCT TCC TG |
| 1 | CYP1B1_3'UTRb | ACG TTG GAT GAG TTG GGT AGT AAC ACT GGG | ACG TTG GAT GTT GTT TGT ACC CTA CAT CGC | CAG GGT ACA TGA TTG TAA CCG |
| 1 | CYP2B6_3'UTRd | ACG TTG GAT GTC CTT GGG CTC CAT GAT CTG | ACG TTG GAT GCA TCT GCA AGA TGG GCA TGA | TGG GGA GAA GAC CCA AGG GAC AT |
| 1 | CYP1B1_3'UTRa | ACG TTG GAT GAG GTA TAC TTC AGT AGA TG | ACG TTG GAT GGA GCA CAC AGG ATC AAA TAC | CAG AAA AGG TTA AAT AAG AAG CTA T |
| 1 | INSL3_311(ex2) | ACG TTG GAT GAC TGA GGC AGC AGT GGC GG | ACG TTG GAT GAT GCT GGT ATT GGC CCC ACA | GCC TCT GGC CAT CAC C |
| 1 | CYP1A2_403(ex1) | ACG TTG GAT GAG GTG TTA AGA GCC TTC TGG | ACG TTG GAT GAT GAC CTT CAA CCC AGA CTC | TCT GGA CAG TGT GGG C |
| 1 | CYP1A2_523(ex1) | ACG TTG GAT GAG GAT CAT GTG AGC AAG GAG | ACG TTG GAT GAT AGG GAT CAA AGT GCC CAG | ATC AGC TCC TGG AAC TT |
| 1 | CYP11A_in7 | ACG TTG GAT GTG CAT CTC CAC TAA AAC CCC | ACG TTG GAT GAC GGT ACA GGT TAA TCC AGC | GCA CCA GGA GAG GGG AT |
| 1 | GUSB_in2a | ACG TTG GAT GAT TGT GGT GAG TGC AGC AAG | ACG TTG GAT GCC AAG GCA GCC ACA TGC GT | GGG CCG TGC CTG TCT GCT |
| 1 | GUSB_in2b | ACG TTG GAT GAG ATG AGG CAA CCC TCC CAA | ACG TTG GAT GAC GTG ACA GCC TGC TTC CTA | GGG CGG CAC TGG GGT TCG TG |
| 1 | CYP1A2_231(ex1) | ACG TTG GAT GAG CAC AGG GGT GCA GCC AAT | ACG TTG GAT GTG CTG ACC TTG GGC AAG AG | CCC TCT ATG GAG ATG TGC TGC A |
| 1 | CYP2B6_3'UTRc | ACG TTG GAT GTT GGG TCT TCT CCC CAA AGG | ACG TTG GAT GTT CTC GGG CTC TGT TTA CTC | TCC CCA TCT GCA AGA TGG GCA TGA TCA |
| 1 | CYP1A2_199(ex1) | ACG TTG GAT GTG CAG CAC ATC TCC ATA GC | ACG TTG GAT GTG CTG ACC TTG GGC AAG AG | CAC ACC TGG CCC TGG C |
| 1 | CYP1A2_508(ex1) | ACG TTG GAT GAT AGG GAT CAA AGT GCC CAG | ACG TTG GAT GTA CCT GGA GGA TCA TGT GAG | ATG TGA GCA AGG AGG C |
| 1 | CYP1A2_329(ex1) | ACG TTG GAT GTG CTC AGC GGC TGG ACA CAT | ACG TTG GAT GAT CAG TGA CCA GAG TGA AGC | GAG TGA AGC TGT GAG GT |
| 1 | PXR_in5d | ACG TTG GAT GTT CAC TGG GCC TCA GCA GTC | ACG TTG GAT GAT TCT CAG TGG CAT GGC CAG | GGG ATT CCC CAT CAC AGC AC |
| 1 | PXR_in4c | ACG TTG GAT GTT CCC AGA CAA GGA AGT GAG | ACG TTG GAT GGG ACT CTG GCT GTT TTC TCC | GCT GTT TTC TCC TAG GGT TG |
| 1 | RASGRP3_3'UTR | ACG TTG GAT GAG ATC CCA CAG TGT CAC AAG | ACG TTG GAT GGA AAC TCT GAA GAA CCC TCC | AAC CCT CCA GCT CTC A |
| 1 | ATP5F1_183(ex3) | ACG TTG GAT GCT GTC ACT CCA GTT TTA GG | ACG TTG GAT GGG AGG AAA AGT TCG TTT GGG | TGG GGC TGA TCC CTG A |
| 1 | ANTXR_3'UTRb | ACG TTG GAT GAC TGC AGC CGT CGG AAA GTA | ACG TTG GAT GGT TGC ATT CTC ACC TGA TAC | CCT AAC CTG ATA CCT GCC ATC |
| 1 | OSBPL6_3'UTR | ACG TTG GAT GGC TTT AAG CAC CAG TTA CAG | ACG TTG GAT GAC ACC CTT CTC GAA AAG CAG | AAA TCA AAA GTC TGA AGA GTC |
| 1 | AKR1C1_144(ex2) | ACG TTG GAT GAT CTT GCT TCG AAT GGC CTG | ACG TTG GAT GGG CCA CCA AAT ATG CCA TAG | GAA GAG AAG TTG GGT TCC GTC A |
| 1 | SPATA7_1460(ex11) | ACG TTG GAT GAG ACT ACC TCG TTT TCG TCC | ACG TTG GAT GCA TTC GAC AGG AAC GTC AAC | GGG AAG GAA CGT CAA CAA TAC CG |
| 1 | TPO_2430(ex13) | ACG TTG GAT GAC GCC ACC CTT GGT GTT CTT | ACG TTG GAT GCA TCA ACG AGT GTG AAG ACG | CGA TAC GGA CCC TCC CTG CCA CGC |
| 1 | DYNC1I2_1123(ex12) | ACG TTG GAT GCA TGT CCA GAC TCC ATG AAC | ACG TTG GAT GGT TGT TGG AAC ACA GAA TGC | CAG CAT GCT CAC AAT CTG ATT AGT |
| 1 | SCP2_3'UTRb | ACG TTG GAT GGT GCA CTA ATG ACC TTC ATC | ACG TTG GAT GGA TGG CAT GAA ATG CAA AT | CCA GTA CAG AAT TTT AAA AAT TCT CA |
| 1 | PLN_-17(prom) | ACG TTG GAT GTT TCA GCT TTC TCT TGA CGG | ACG TTG GAT GTA ATA GCA GAG CGA GTG AGG | GAT ACC AGG AAG GCA G |
| 1 | BCKDHB_3'UTR | ACG TTG GAT GCC ATT AAG AGT ATT TCA GAG | ACG TTG GAT GCC CAC AGC CAA TAT GTA CAG | CCT ACC ATG TGG AAC ATT T |
| 1 | GSTO1_510(ex5) | ACG TTG GAT GCT TCC AGC CTT TCA AAC CAG | ACG TTG GAT GGA AGA CAA CAT ACT TTG GTG G | TAC TTT GGT GGA AGT TCT CT |
| 1 | PHYH_297(ex3) | ACG TTG GAT GAC ATT GTT TGT GCC TGG ACG | ACG TTG GAT GTG AGT TCC TGG GAA CAC AAC | CCC CCA GCC ATT TTT CCG GTC |
| 1 | CDH2_2655(ex16) | ACG TTG GAT GAG CGA GCT TCT TGA AAC GTG | ACG TTG GAT GAG CTC CCT TAA TTC CTC CAG | GTG TAG GTG AGC AGG ACT ATG A |
| 1 | SC4MOL_3'UTRa | ACG TTG GAT GAC TGG TCA GCT CCA GAT AAG | ACG TTG GAT GTC CAT ACA GTG TGA GTG AGG | GGC GGC CGA ATC CGA AAT AGC CA |
| 1 | EP300_3'UTR | ACG TTG GAT GCT ATT CCA TAA GTC TGA GCG | ACG TTG GAT GAG CTG CCC CTG TAT ATT TTG | TTT CTC TCT ACA TGT ACA AAT TAC |
| 1 | ZF_3'UTR | ACG TTG GAT GAC CAC AGA AAC TAG TAT ACC | ACG TTG GAT GTG GCT TGA GCT TTC TGT GAG | GGA CTT CTA AAA AAT ATT TTG AGG |
| 1 | GBE1_3'UTR | ACG TTG GAT GGA TAA AGG AGA ATA ACA GTC | ACG TTG GAT GAT GGC AGA TAT TCT ATG AC | AAG GTC TCT TGG TCT AAA TAA CAG A |
| 1 | PGRMC1_3'UTR | ACG TTG GAT GTC GTG TGA AAG AAC CAC TGC | ACG TTG GAT GGG CTT GCC TAG AAA CCG AAA | GGG AAC GAA ATA TAC GAG GAA GAT TG |
| 1 | EFEMP1_3'UTR | ACG TTG GAT GCT GGT CTT CTT CAA GAG AGC | ACG TTG GAT GGC TTG CTC CCA CTT TTA CAG | CCC CTA ATC CAA GTT TCA TTG TAT AGA |
| 1 | LARP7_1233(ex8) | ACG TTG GAT GGT ACT CCA TGG TCT GTT TCC | ACG TTG GAT GCA AAA AGC CAG CAT GGC TTC | AAA AAA AAG AAT ATC CCA AAT AAA ATC |
| 1 | TMEPAI_3'UTRa | ACG TTG GAT GAT GCA GCG TGT CCT TCT GAG | ACG TTG GAT GTC GTT GGG TTT GGT TTT CCG | AAC TGC GCT TCA AAT G |
| 1 | GOLPH3_828(ex3) | ACG TTG GAT GTA CGA GAG GCC TTC TTT CTG | ACG TTG GAT GCA GTG AAG ATG GCA ACA AGG | CAG CAA CAA GGA GTG GAA |
| 1 | RLN_12(ex1) | ACG TTG GAT GAA GAG ATC AGG TCC AGG ATG | ACG TTG GAT GTT TCT CTG GGA AGT TGG CTC | TTC CCT AGG AGG TAG GAG AAC |
| 1 | AKR1C1_768(ex7) | ACG TTG GAT GAA AGC ACA AGC AAA CCT CAG | ACG TTG GAT GTT GTA ACT CTT GGC CAG GAC | GGG GTG CAG CTG GTA GCG AAG |
| 1 | IDI1_884(ex5) | ACG TTG GAT GGG GTG AAG TTA AGG TGA CTC | ACG TTG GAT GCC CAC CAG TGA AAG AGA AAC | CAG CGA AAC CTA TCT GCA ATC AT |
| 1 | AKR1C1_36(ex1) | ACG TTG GAT GCA GGT GAT GGA TCC CAA AAG | ACG TTG GAT GGC ATA GGT ACC AAA TCC CAG | GAA GGT ACA GGA ATG AAG TGA CC |
| 1 | TEGT_3'UTRa | ACG TTG GAT GTG GGC ACT AAT AGT GTC AGG | ACG TTG GAT GCC TAG AGA ACA GGT GAA CAG | CCC ATG CAT CTA TAA ACA GAC ATC |
| 1 | NR5A2_3'UTR | ACG TTG GAT GAG GCA GTA ATT TCC TTA ACG | ACG TTG GAT GCC CGC TTT TGA TTT TAG TGT | CCC ACT TTT GAT TTT AGT GTC TCA CG |
| 1 | ANTXR_3'UTRa | ACG TTG GAT GCT TCT TTA GAA TAG TCC CCC | ACG TTG GAT GTG CAG GTG ACC AAG ACA TTC | TGC TTC ATG ACC CCT G |
| 1 | TIMM17A_3'UTR | ACG TTG GAT GAC GGT TTT CAA AAC CGA CTG | ACG TTG GAT GCA GTA GGA CTT CTT TCC CAG | ACG TGG TTC AAG GAG GGA |
| 1 | TIMM17A_252(ex4) | ACG TTG GAT GCT CGT GAT GGA GTT CCA GG | ACG TTG GAT GGG AGG CCT GTT TTC CAC GAT | GGA GGC ATG GCC CAG GTG CG |
| 1 | XPOT_2694(ex21) | ACG TTG GAT GAC TGA ACA CAT TCT GGA CCC | ACG TTG GAT GGA TGC ACA AAC AGT ATT GGC | GCA CAA ACA GTA TTG GCT TTA TC |
| 1 | ME1_3'UTRb | ACG TTG GAT GAT GAA AGA CTT CCA GAT C | ACG TTG GAT GAG GCC CAG AAA ATA CAG ACC | TCA TAG CCT ACA TTT CTA ACT CCA |
| 1 | PIK3R1_3'UTRa | ACG TTG GAT GGA AGA AAT GTA CAG GAT GC | ACG TTG GAT GAG CCA AGT ACT CTG TAC AAG | GAA TTC TGA AGC CAT ATT TTT TTT |
| 1 | LARP7_1362(ex9) | ACG TTG GAT GTT GTT TCC TGC CAG GTA GAG | ACG TTG GAT GGG TTA ATG CAA CAG GAC CTC | GGT GGG ACC TCA GTT TGT GAG CGG |
| 1 | RLN_370(ex2) | ACG TTG GAT GCT TCT GCT TCA TTT TGT CTG | ACG TTG GAT GAG AGA GCT ACA ACA ATC TGC | AAT GAA AGG ATT CGA ATC TTA ACT T |
| 1 | TES_3'UTR | ACG TTG GAT GGA TAC ATG GTG AAT TCC TGT G | ACG TTG GAT GGG AAA TAG CAA ATG CAT AGG | GTT GTC ATA CTT GCT GCA TTT CTA TCT |
| 1 | PSMA7_157(ex2) | ACG TTG GAT GAT GCA GAC GTT GTC GTC CAG | ACG TTG GAT GCG TGG AGA AGA AGT CGG TG | GAA GTC GGT GGC CAG G |
| 1 | EMILIN2_3'UTR | ACG TTG GAT GGC AGC TAC ACA CAC AAA CTG | ACG TTG GAT GAT CCT TTC CTT TCC CAC CTC | CTG TCA GAA CTC AGC AG |
| 1 | PSMA7_3'UTR | ACG TTG GAT GAA TCA CTG CGA GCG AGC TTC | ACG TTG GAT GGA AGT ACG TTG CTG AGA TTG | GGA AGC AGA AGA AGG CG |
| 1 | TMEPAI_3'UTRb | ACG TTG GAT GGG AGC AAC GTC AAA GCG TC | ACG TTG GAT GAT AGA GCT CAG AAG GAC ACG | GAT TCC ACG GTG GTT GTG C |
| 1 | NR4A2_3'UTR | ACG TTG GAT GAA TGC CCT TTC AGG TTC TGC | ACG TTG GAT GCC AGC CCT TTG ATC CCT AAA | CCT TTG ATC CCT AAA GAA AAA |
| 1 | RHOB_3'UTR | ACG TTG GAT GAG GGA GAA AGG AGA CAC GAG | ACG TTG GAT GGC AGT CTC TAA AGC TGT GTG | GAG GCA TCT CTG TAC AGA GAA |
| 1 | SPATA7_1795(ex11) | ACG TTG GAT GAG TCC TCA ATG CTC ATT TCC | ACG TTG GAT GAG CAC TCT CTC CAT CTG TTC | ATC AGT GGT GAT AAT CAG AAC A |
| 1 | EMILIN2_3250(ex8) | ACG TTG GAT GGT CAA CAT CGT GGT GAC CG | ACG TTG GAT GGA AAA CCC CAC TAA ACG TTG | CCA ATC TCA TCA AAG TCC GTG TG |
| 1 | RLN_261(ex1) | ACG TTG GAT GTG TTG CCT TCA GCT CCT GTG | ACG TTG GAT GAC CAT GCC ATC CTC CAT CAC | GAG GCA GAA ATC TTA AAG ATG ATG T |
| 1 | ME1_3'UTRa | ACG TTG GAT GGT CTA CTG TGA TGT TTA CCC | ACG TTG GAT GGA TAA TTT CCC CTT AAC AC | TGG GTA ATT TCC CCT TAA CAC TCT AAA |
| 1 | TIMM17A_285(ex4) | ACG TTG GAT GTT CTT GCT GCC AGG ATG GCT | ACG TTG GAT GAG GAC CCC TGG AAC TCC AT | CTG GAA CTC CAT CAC GAG |
| 1 | SCP2_3'UTRa | ACG TTG GAT GAA AAT GGG TTT CCT TCT TG | ACG TTG GAT GCA ATT ATT TTT CAC AAC AGT G | CAA CAG TGT TTT ATG ATC CC |
| 1 | PIK3R1_3'UTRb | ACG TTG GAT GAT GGC TTC AGA ATT AAA AC | ACG TTG GAT GCC CCA AAA TCT GTT ATG AAC C | AAC CTT TTA ACC TTG GGC TAA |
| 1 | SCP2_3'UTRc | ACG TTG GAT GTA GAT GAA GGT CAT TAG TGC | ACG TTG GAT GAA GTT TTC CTT GGG AGG CCG | GAG CAC GAA CAC CAG TAG TGG |
| 1 | CALCA_3'UTR | ACG TTG GAT GCT CAC AGA CAA GGT TAG AGG | ACG TTG GAT GTG AAG CAG GAA TTG GAG GAC | ACA TGA TCT CAG AAG CTT CCC T |
| 1 | XPOT_2709(ex21) | ACG TTG GAT GGA TGC ACA AAC AGT ATT GGC | ACG TTG GAT GAC TGA ACA CAT TCT GGA CCC | GTT TGA GAT GAA TTG TTT TCA G |
| 1 | TEGT_3'UTRb | ACG TTG GAT GGA GTT ACA TCT CTT GAA GCC | ACG TTG GAT GGT CAT TCT TGG CAC TGT TTC | GCA CGC AAA AAA AAA GCA CAA GA |
| 1 | ZNF514_3'UTR | ACG TTG GAT GAC CTT TAT GAG GCA GTG GAG | ACG TTG GAT GCT AGT AGC ATA AAG ATC TC | GGG GCT CTA AAA TTG CTT TTT TGG T |
| 1 | GOLPH3_3'UTR | ACG TTG GAT GGT ACG ATT TAG CCA CCA AGC | ACG TTG GAT GAT AGC ACC TCG TTG GTG CTG | TTC AGA CAC TCC ACT TC |
| 1 | CYP3A29_1180(ex9) | ACG TTG GAT GGT AAG TGG AGC CTG ACT TTC | ACG TTG GAT GTA AAT CTC TGG TGC TCT GGG | GCT CTG GGA TGC AGC TT |
| 1 | IGFBP7_3'UTR | ACG TTG GAT GAA AGT TCC TTC TAC AAG TCC | ACG TTG GAT GCC AGT GTA AGT TTT ACC TTA G | CTT AGA ATT CCT CTT GGC T |
| 1 | SC4MOL_3'UTRb | ACG TTG GAT GGC AAG TCA GTT CAC AAA GTC | ACG TTG GAT GAA TTG AGC TTT ATT TCT GC | TAA CTA CCC TTT TGT TTT TGT |
| 1 | EMILIN2_3163(ex8) | ACG TTG GAT GAG AAT TCC TGG AGT ACC ACC | ACG TTG GAT GAA GTG CAC GAC CAG GTG GAA | GGA AGG CGC CTG GGC CCC CGC A |
| 1 | NCOA2_3'UTR | ACG TTG GAT GTT CGT TTT CCC TAG CTA ACC | ACG TTG GAT GGA ATT CGG CAC GAG GGT TTA | ATT TTT TTT TTT TGA AAT TCT TTT CT |
